# The impact of alterations in lignin deposition on cellulose organization of the plant cell wall

**DOI:** 10.1186/s13068-016-0540-z

**Published:** 2016-06-17

**Authors:** Jiliang Liu, Jeong Im Kim, Joanne C. Cusumano, Clint Chapple, Nagarajan Venugopalan, Robert F. Fischetti, Lee Makowski

**Affiliations:** Department of Bioengineering, Northeastern University, 360 Huntington Ave, Boston, MA 02148 USA; Department of Biochemistry, Purdue University, 175 South University Street, West Lafayette, IN 47907 USA; GM/CA CAT, XSD, Advanced Photon Source, Argonne National Laboratory, 9700 Cass Ave, Lemont, IL 60439 USA; Department of Chemistry and Chemical Biology, Northeastern University, 360 Huntington Ave, Boston, MA 02148 USA

**Keywords:** Lignin biosynthetic mutants, Cellulose fibril, X-ray microdiffraction

## Abstract

**Background:**

Coordination of synthesis and assembly of the polymeric components of cell walls is essential for plant growth and development. Given the degree of co-mingling and cross-linking among cell wall components, cellulose organization must be dependent on the organization of other polymers such as lignin. Here we seek to identify aspects of that codependency by studying the structural organization of cellulose fibrils in stems from *Arabidopsis* plants harboring mutations in genes encoding enzymes involved in lignin biosynthesis. Plants containing high levels of G-lignin, S-lignin, H-lignin, aldehyde-rich lignin, and ferulic acid-containing lignin, along with plants with very low lignin content were grown and harvested and longitudinal sections of stem were prepared and dried. Scanning X-ray microdiffraction was carried out using a 5-micron beam that moved across the sections in 5-micron steps and complete diffraction patterns were collected at each raster point. Approximately, 16,000 diffraction patterns were analyzed to determine cellulose fibril orientation and order within the tissues making up the stems.

**Results:**

Several mutations—most notably those exhibiting (1) down-regulation of cinnamoyl CoA reductase which leads to cell walls deficient in lignin and (2) defect of cinnamic acid 4-hydroxylase which greatly reduces lignin content—exhibited significant decrease in the proportion of oriented cellulose fibrils in the cell wall. Distinctions between tissues were maintained in all variants and even in plants exhibiting dramatic changes in cellulosic order the trends between tissues (where apparent) were generally maintained. The resilience of cellulose to degradative processes was investigated by carrying out the same analysis on samples stored in water for 30 days prior to data collection. This treatment led to significant loss of cellulosic order in plants rich in aldehyde or H-lignin, less change in wild type, and essentially no change in samples with high levels of G- or S-lignin.

**Conclusions:**

These studies demonstrate that changes in lignin biosynthesis lead to significant disruption in the orientation and order of cellulose fibrils in all tissues of the stem. These dramatic phenotypic changes, in mutants with lignin rich in aldehyde or H-units, correlate with the impact the mutations have on the enzymatic degradation of the plant cell wall.

**Electronic supplementary material:**

The online version of this article (doi:10.1186/s13068-016-0540-z) contains supplementary material, which is available to authorized users.

## Background

Plant cell walls are complex structures composed largely of high molecular weight poly-saccharides, highly glycosylated proteins, and lignin [1]. Little is known about the interdependence that the assembly of different polymers have on one another. That there are codependencies among the synthesis and assembly of different polymeric species seems obvious given the degree of co-mingling and cross-linking intrinsic to the cell wall, but the details of those dependencies are largely unknown. Examination of the phenotype of three mutations that cause collapse of mature xylem cells in inflorescence stems of *Arabidopsis* [2] suggested that a normal pattern of cellulose deposition may be required for assembly of lignin. Disruptions in lignin metabolism that result in changes in the final lignin composition of cell walls may lead to changes in both microscopic and macroscopic morphologies as well as changes in digestibility [3, 4].

Dissection of the lignin biosynthetic pathways [5] has been enabled by the identification of numerous *Arabidopsis* mutants with altered lignin compositions and disrupted cell wall microstructure. Lignin deficiency may be associated with dwarfism, breakdown of vascular tissues, alteration of microstructure, but the relationship between lignin content and plant size and recalcitrance is complex. For instance, disruption of the Mediator complex subunits MED5a (*med5a*) and MED5b (*med5b*) rescues the stunted growth, lignin deficiency, and changes in gene expression seen in the phenylpropanoid pathway mutant *reduced epidermal fluorescence8* (*ref8*) without restoring the synthesis of guaiacyl and syringyl lignin subunits [6]. The lignin in the resulting *med5a med5b ref8* triple mutant is almost entirely H-lignin and the yield of glucose on treatment with a mixture of commercial cellulase and β-glucosidase enzymes was significantly greater than in wild type [6]. The increased susceptibility to enzymatic digestion is suggestive of molecular-level alterations that increase access of enzymes to cellulose within these plants and suggests that in addition to changes in lignin composition and content, cellulose structures may be altered.

Multiscale imaging using light and electron microscopy can provide information about changes in the overall organization of the cell wall but limited information about changes in the molecular-level architecture. X-ray scattering represents a powerful approach to studies of alterations in the molecular-level structure and organization of cellulosic material in plant cell walls [7–9]. X-ray patterns derived from intact tissues such as the *Arabidopsis* stem contain the superposition of scattering from all constituents. Interpretation of the scattering is usually limited to insights about the organization of constituents that exhibit well-characterized scattering patterns that can be readily identified and quantitated in the context of a varying background made up of scattering from all other components. X-ray scattering from cellulose is very well characterized and readily quantified. Conversely, scattering from lignin, hemicellulose, and other cell wall components is not well defined and usually constitutes diffuse, unoriented scattering that is treated as background beneath the better defined scattering from cellulose. Consequently, interpretation of scattering from *Arabidopsis* stems is usually confined to conclusions about alterations in the orientation and organization of the cellulose.

Direct analysis of the impact of mutations on cellulosic structures is complicated by the architectural heterogeneity among different tissues which may react differently to changes in lignin composition and deposition. Tissues in the *Arabidopsis* stem may be only a few 10’s of microns across. Any method that averages structural information over larger areas runs the risk of missing tissue-specific variables. Differences in cellulosic structures in these tissues can be distinguished by the use of an X-ray microbeam a few microns in diameter [9–12]. Analysis of the cellulosic structure within *Arabidopsis* stem has been carried out by scanning X-ray microdiffraction (SXMD) using a 5-micron diameter X-ray beam scanned across a thin section of stem in 5-micron steps collecting a full-diffraction pattern at every pixel [9]. This strategy proved effective at differentiating the organization of cellulose within the different tissues making up the wild-type stem, providing information on the orientation and size of microfibrils and the relative abundance of amorphous materials and crystalline (fibrillar) cellulose. Here, we describe experiments using this same strategy to characterize differences in cellulose organization within plants harboring mutations in lignin biosynthetic genes. The results demonstrate that cellulose organization in *Arabidopsis* is often significantly impacted by changes in lignin composition and synthesis.

We collected SXMD data on wild-type *Arabidopsis* and 7 variants, each with a well-characterized alteration in some aspect of lignin biosynthesis. Lignin in wild-type plants typically contain primarily guaiacyl (G) and syringyl (S) monomeric units, with H-monomers accounting for <2 % of total lignin. Variants studied here are as follows:

1. [*fah1*-*2*] A ferulic acid hydroxylase 1 (*fah1*-*2*) mutant that is blocked at ferulate 5-hydroxylase (F5H) and fails to deposit S-lignin [13] [14] resulting in a plant that deposits primarily G-lignin [*fah1*-*2*] resulting in plant cell walls less digestible than wild type or walls high in S-lignins [4].
2. [*C4H::F5H fah1*-*2*] An F5H overexpressing transgenic plant *C4H::F5H fah1*-*2* [15] that deposits mostly S-lignin and grows like wild type but exhibits a substantially greater susceptibility to cellulases than wild type [4, 15, 16]. Maleic acid-treated material from these plants exhibits microstructure breakdown with fiber cells disjoined from each other, suggesting an origin of their high susceptibility to digestion [4].
3. [*med5a med5b ref8*] A triple mutant *med5a med5b ref8* containing predominantly H-lignin [*med5a med5b ref8*] [6].
4. [*cadc cadd*] A plant-harboring lesions in both *cadc* and *cadd* leading to the incorporation of coniferyl and sinapyl aldehydes in place of their corresponding alcohols [3, 17]. These plants produce about 50 % the level of total lignin seen in wild-type plants and display a limp floral stem and collapse of xylem elements in spite of total cellulose not being significantly different from wild type.
5. [*cadc cadd fah1*] A triple mutant, *cadc cadd fah1*, that synthesizes lignin dominated by guaiacyl-substituted aldehyde units and contains total lignin about half that of wild type [3].
6. [*ccr*] A plant with down-regulation of cinnamoyl CoA reductase 1 (*ccr*—the first enzyme specific to the biosynthetic pathway leading to monolignols), a genetic perturbation which gives rise to cell walls rich in cell wall-bound ferulic esters and an improvement of cell wall digestibility [18–20].
7. [*ref3*-*2*] A *ref3*-*2* mutant with a missense mutation in the gene encoding cinnamic acid 4-hydroxylase (*C4H*) resulting in reduced lignin deposition and altered lignin monomer content. Plants harboring this defect exhibit collapse of vessel elements [21].

Here we investigate the molecular architecture of cellulose within thin sections of stems from these variants to determine if the alterations in lignin biosynthesis lead to changes in the organization, orientation or order of cellulose fibrils. These studies indicate the structure of cellulose fibril may be disrupted in *ref3*-*2* and *ccr1*, and the digestibility of plants harboring lesions in *cadc cadd*, *cadc cadd fah1*, and *med5a med5b ref8* may be greater than wild-type plants.

## Results

### Scanning microdiffraction of longitudinal sections of *Arabidopsis* stem

Scanning X-ray microdiffraction of longitudinal sections of stems from wild type and seven mutants of *Arabidopsis* was carried out in order to assess fibril orientation and organization in the tissues of the stem. Approximately, 16,000 diffraction patterns were collected on 32 samples. Samples were harvested and stems microtomed into 100-micron-thick longitudinal sections and stored in water (either 3 days or 30 days) until just prior to data collection at which point they were dried in air for 24 h. Analysis of two repeats of fresh and stored samples resulted in essentially identical results in experiments carried out over 2 years.

**Fig. 1:**
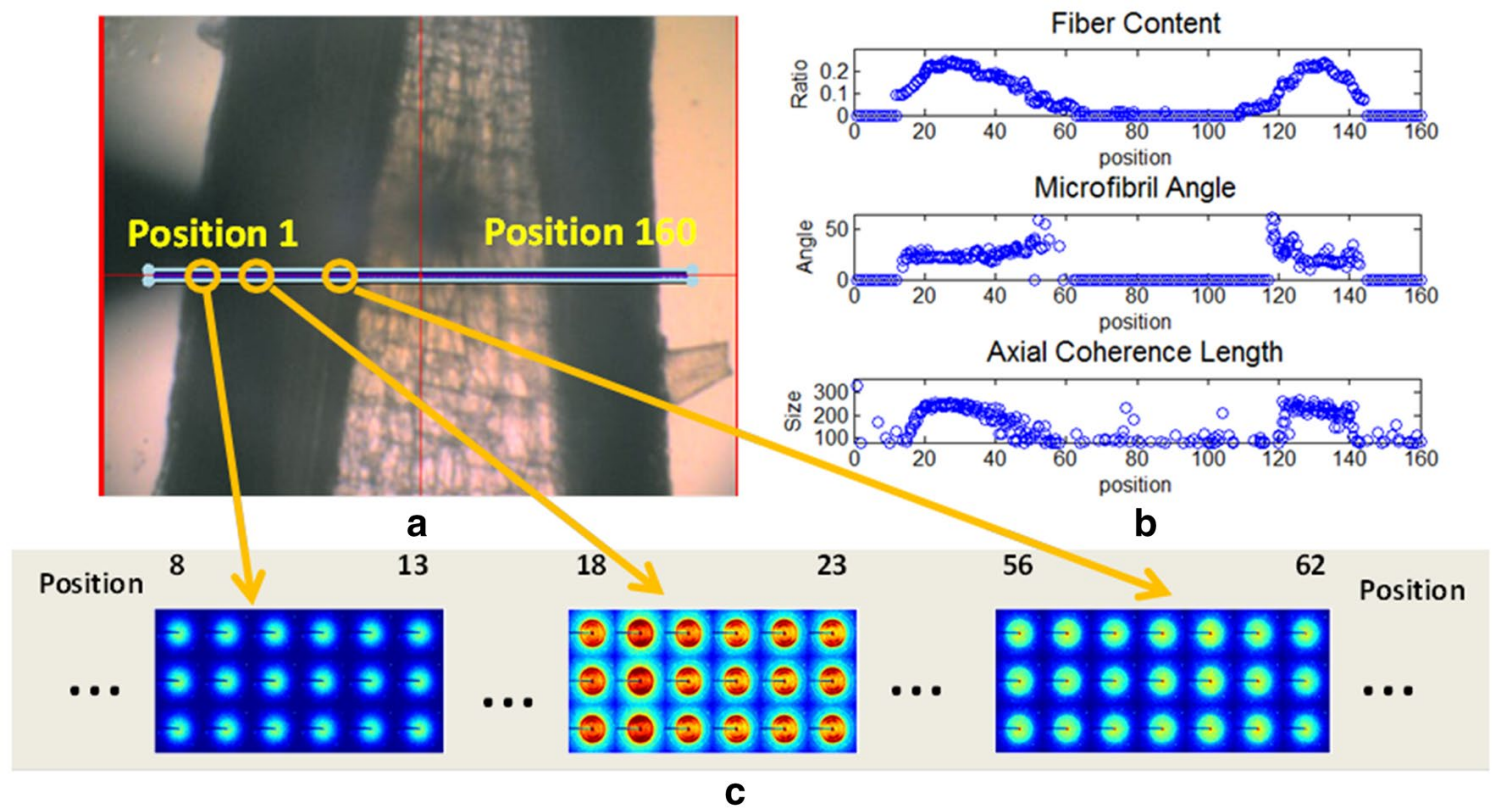
Scanning X-ray microdiffraction data from a longitudinal section of *Arabidopsis* stem. **a** Optical image of the stem with the region scanned marked as a *blue rectangle*. The grid includes three rows and 160 columns, each grid point being 5-microns square, making the grid 800 × 15 microns. A complete microdiffraction pattern was taken at each grid point. **b** Fiber content, microfibril angle, and axial coherence length as calculated from the diffraction patterns collected by scanning the* rectangle* in (**a**) and plotted as a function of position across the stem. Data from all* three rows* are plotted. **c** Enlargement of microdiffraction patterns from selected regions of the scan. Selected regions correspond roughly to the cortex, xylem, and close to pith

A 5-micron X-ray beam was scanned over a grid with 5-micron step size, and a diffraction pattern was collected at each grid point. The scattering angles subtended by the patterns ranged from a spacing of ~100 Å at the beam stop (small-angle regime) to ~2 Å at the detector edge (wide-angle regime). In most cases, a 300 mm sample to detector distance was used. Figure 1 provides an example of data collected from a thin section of wild-type *Arabidopsis* stem. Samples were aligned using a coaxial optical microscope with a hole down the optical axis to accommodate the X-ray beam thereby allowing precision alignment of the beam to select positions on the sample. Figure 1a shows an optical micrograph of the wild-type sample with a blue rectangle marking the position of a 160 × 3 grid of positions from which diffraction patterns were taken. Figure 1c contains 57 diffraction patterns selected to represent data from the cortex, xylem, and the region adjacent to the pith.

Software was developed to automatically extract specific structural information from these patterns (see “Methods” section for details). A number of structural parameters were extracted from each diffraction pattern and their variation as a function of distance across the stem mapped. Figure 1b includes the distribution of fiber content, microfibril angle, and axial coherence across the sample pictured in Fig. 1a. Each of these parameters is described in more detail below. The distributions represent a mapping across the entire breadth of the stem, starting with the epidermis, moving to the vascular tissues and then the pith, and continuing to reverse this sequence to the other side of the stem. The organization of tissues in *Arabidopsis* stems is not circularly symmetric about the step axis, and within the 100 micron thickness of the sections used, there is an occasional superposition of regions from different tissues. The cambium, the region between xylem and phloem appears to correspond to the region with the greatest fiber content, consistent with our earlier observations [9]. From the mapping of specific properties, representative parameters were extracted to ease the comparison of structural order present in the different variants.

### Oriented fiber content

We have observed that the proportion of material exhibiting orientation varies widely among the mutants studied here (as well as among different tissues within individual stems). In order to quantitate this variation, we chose to calculate a measure of the proportion of total material made up of oriented cellulose fibrils. As implied by the results summarized in Fig. 12, not all cellulosic material is well oriented. But the vast majority of oriented scattering can be attributed to cellulose fibrils. Consequently, the ratio of oriented intensity to disoriented intensity provides a relative measure of the extent of structural organization of cellulose fibrils within the scattering volume. As shown in Fig. 1b, oriented fibril content is the largest in the vascular regions, particularly the xylem, and essentially zero in the pith and epidermis where no scattering attributable to oriented, fibrillar cellulose is observed.

**Fig. 2:**
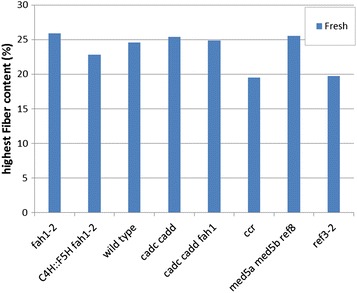
Comparison of the highest oriented fiber content observed in each sample. The *blue bars* correspond to the dried samples 3 days after harvest

Variations of oriented fiber across the stem for each mutant and wild type have been exhibited in Additional file 1: Figures S1–S8. In order to compare the proportion of oriented fibrils in the different variants, we focused on the cambium the region between xylem and phloem which exhibits the highest content of oriented fibrils [9]. Figure 2 shows a bar graph which compares the maximum values of oriented fiber content observed in each of the eight samples (utilization of average values—rather than maximum—results in a plot exhibiting the same trends). Figure 2 also shows that only *ccr* and *ref3*-*2* exhibit significant reduction of fiber content. *ref3*-*2* and *ccr* are the samples with reduced lignin content [21] suggesting that assembly of cellulose fibrils into oriented structures parallel to the stem relies on the presence of normal levels of lignin. *ccr* has high levels of ferulic acid-containing cell walls and is more digestible than wild type.

### Crystalline order in the cellulose fibrils

**Fig. 3:**
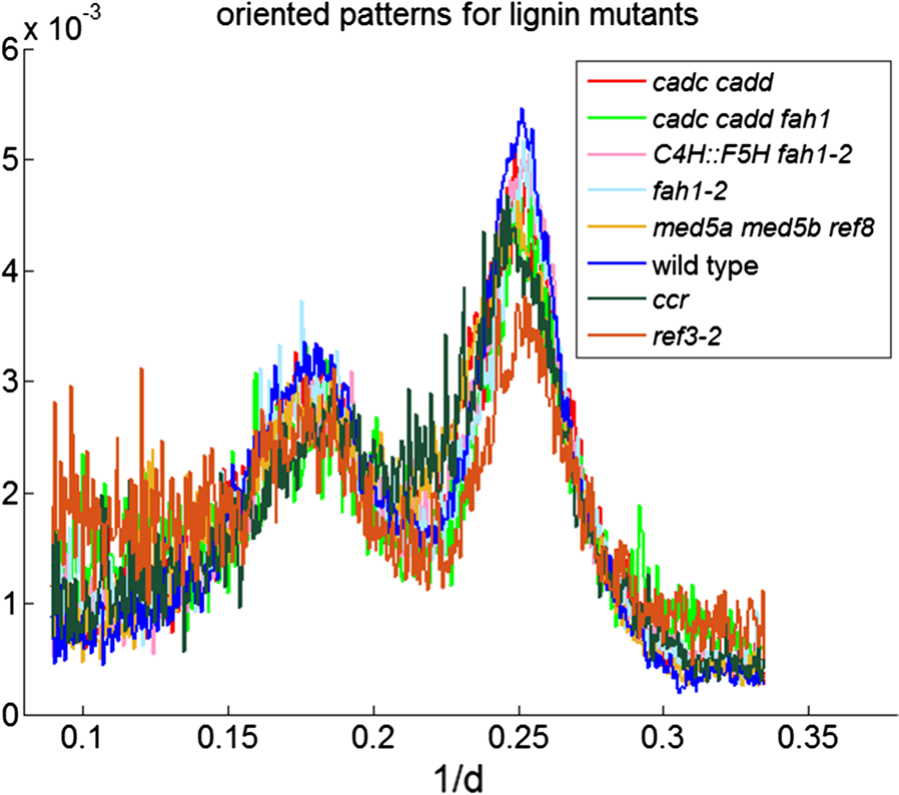
Traces of the intensities of the strongest equatorial reflections of from cellulose Iβ in the eight *Arabidopsis* variants studied here. In each case, the trace corresponds to that position in the sample that exhibited the highest proportion of oriented cellulose fiber content. Intensities were normalized over the range 0.1–0.3 Å^−1^ for comparison

The positions of the (1 1 0)/(1 −1 0) and (2 0 0) reflections of cellulose provide a measure of the lattice dimensions in the directions perpendicular to the fibril axis. The widths of these reflections are determined by a combination of crystallite breadth (number of cellulose chains in the fibril), cross-sectional shape, degree of order in the packing of cellulose chains, and heterogeneity of crystal lattice constants. The difficulty in separating these three variables is a key reason for the continued debate over the number of cellulose molecules making up an elementary fibril. Nevertheless, the sharpness of these peaks provides a measure of the homogeneity of crystalline order within the cellulose fibrils averaged over the scattering volume. Figure 3 shows the traces of the (1 1 0)/(1 −1 0) and (2 0 0) reflections in scattering from the different lignin mutants. Each trace comes from diffraction pattern exhibiting the highest fiber content for corresponding mutants. The pattern corresponding to the highest fiber content from each sample is compared here for the eight samples analyzed 3 days after harvesting. The (2 0 0) reflection is broadest for *ccr* and *ref3*-*2*, the samples with reduced lignin content. Analysis of Raman spectrum showed that either disorganization or size of crystalline cellulose fibril within plant cell could lead to variation of half width of (2 0 0) reflections [22]. Whether this is due to intrinsic disorder within individual fibrils or to a structurally heterogeneous population of fibrils cannot be determined with existing data. However, these also have the lowest crystalline cellulose content suggesting that lowered lignin content leads to both a decrease in the fraction of crystalline cellulose and the degree of order intrinsic to the crystalline cellulose fibrils.

### Microfibril angle

**Fig. 4:**
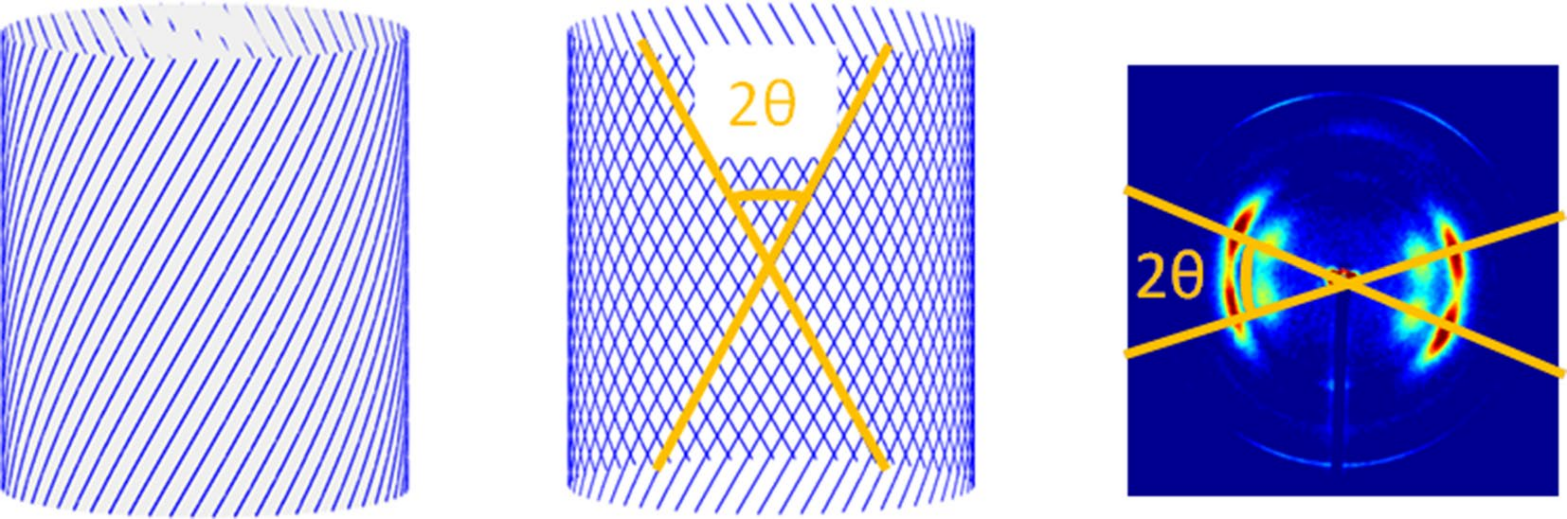
Depiction of cellulose fibrils helically wrapped around a plant cell (*left*). Projection of cellulose fibrils from front and back produce a double orientation (*center*) that expresses itself in diffraction patterns as a split or double diffraction pattern (*right*)

**Fig. 5:**
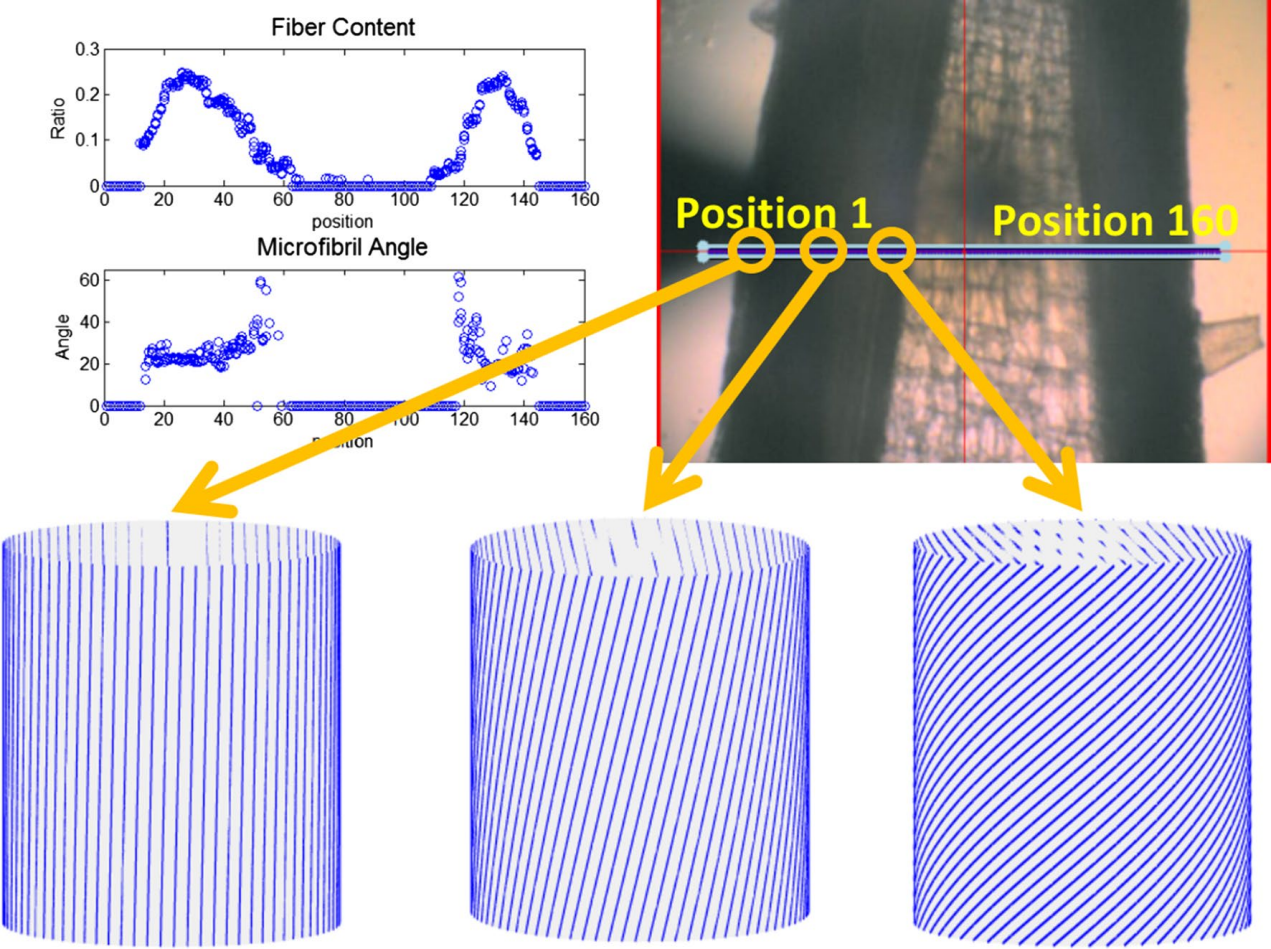
The microfibril angle increases from the periphery of the stem to the center, reflecting a change in the way cellulose fibrils coil around cells. Microfibril angle is the greatest in regions of lowest oriented fibril content as can be seen in the plots in the *upper left*. The grid within the optical image shows the region scanned and identifies positions with fibril orientation corresponding approximately to the diagrams at the bottom of the figure

X-ray patterns from many cellulose containing plants exhibit a double orientation—they appear as two diffraction patterns superimposed and rotated relative to one another. The explanation for this observation is the helical winding of cellulose about plant cells that lead to different orientations of the cellulose on the near and far side of a cell [23] as diagrammed in Fig. 4. Microfibril angle was calculated by transforming all intensity onto a polar coordinate system and identifying angles of maximum intensity as detailed in “Methods” section. Because of the symmetry of scattering from cellulose, two independent measures of microfibril angle are obtained for each pattern and these were averaged. In many cases, the diffraction was almost circularly symmetric and no microfibril angle could be determined. Figure 1b shows the distribution of microfibril angle for the wild-type sample. Figure 5 shows that the helical winding of cellulose in the cortex and outer part of vasculature tissue has relatively constant microfibril angles of about 15°, but the angle gradually increases across the inner part of the vascular bundles and fibers to about 30° immediately adjacent to the pith. The lower microfibril angle correlated with observation from confocal Raman microscopy is consistent with the observations of Gielinger [24] and Mateu et al. [25] who reported that orientation of cellulose tends to be parallel to the elongated direction at outer xylem and cortex. Orientation was seldom observed in the pith, precluding an estimate microfibril angle. For many of the mutants with severe reductions in lignin content, orientation is so poor as to preclude measurement of microfibril angle. *C4H::F5H fah1*-*2* mutants exhibit a microfibril angle distribution similar to fresh samples. The trends of microfibril angle for other samples are less clear. See Additional file 1 for details.

 Microfibril angle tends to be inversely related to oriented fiber content within individual samples. In a comparison of wild-type and the lignin variants, there is no well-defined correlation between microfibril angle and fiber content. Measurement of microfibril angle is in some cases precluded for mutants with severe reduction of lignin content. As shown in Figs. 4 and 5, the microfibril angles display the tilt to longitudinal direction of cell, larger microfibril angle indicates cellulose fibrils were more transversely arranged within the cell wall. For instance, only small portions of the *ref3*-*2* mutant stem exhibit diffraction patterns with the split reflections required to measure microfibril angle. The mean microfibril angle in the xylem of *ref3*-*2* is about 25°, which is greater than that for wild type and *C4H::F5H fah1*-*2* and suggests that the transverse assembly of cellulose is altered in *ref3*-*2*.

It had been well established that in the interfascicular or vascular tissues of *Arabidopsis* stem, the deposition of lignin decreases as one approaches the pith [26]. Decreasing lignin concentration correlates with decreased fiber content and increased microfibril angle, but to what extent there are causal relationships among these three variables is unclear.

### Axial coherence length

**Fig. 6:**
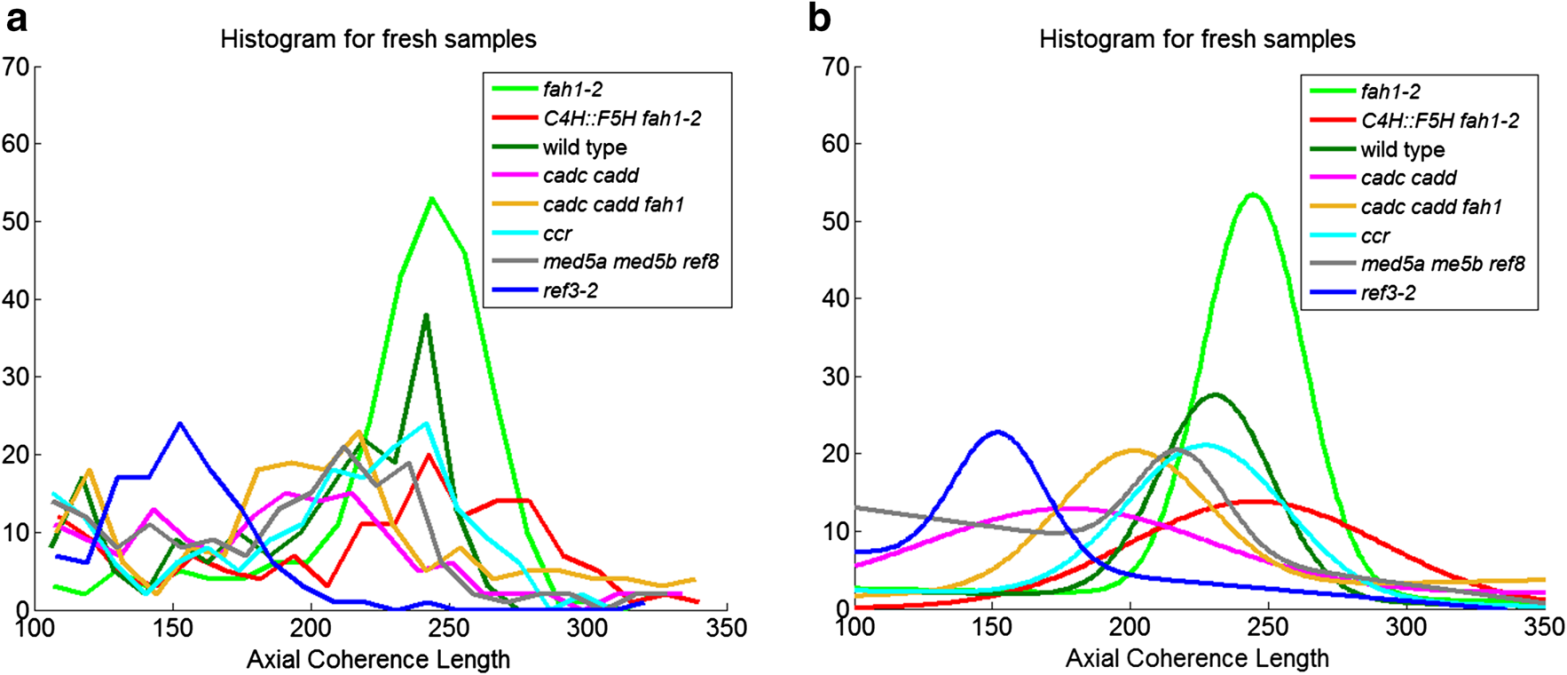
**a** Histogram of axial coherence length for samples dried fresh. The axial coherence length differs across the stem, tending to be largest in the vascular tissues. **b** Gaussian curves fit to each of the histograms in **a** suppress the effect of random variations and facilitates visual comparisons among the samples

**Fig. 7:**
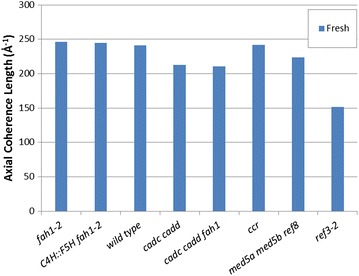
Comparison of maximum coherence lengths for each of the right samples. This is a compilation of the peak positions of the smoothed curves shown in Fig. 8b

A key measure of the crystallinity of cellulose is its axial coherence length. In the axial direction, a useful measure of the degree of imperfection is the coherence length as estimated by the breadth of the (0 0 4) reflection in fiber diffraction patterns. Coherence length may vary among the tissues of the stem as detailed in the figures in the Additional file 1. For simplicity, we chose to compare the distribution of axial coherence lengths observed for each of the lignin mutants as shown in the histograms in Fig. 6. Figure 7 provides comparison of the maximum coherence lengths observed for each of the samples. In fresh samples, *ref3*-*2* and samples with high content of aldehydes ([*cadc cadd fah1*-*2*] and [*cadc cadd*]) appear to have a somewhat lower coherence lengths, with *fah1*-*2* and *C4H::F5H fah1*-*2* exhibiting slightly higher average coherence lengths than wild type.

Coherence length of a cellulose fibril may be influenced by the cross-links it makes with other cell wall constituents. Cross-links may not be easily accommodated by a highly regular crystalline structure. This suggests that coherence length might provide a measure of the degree to which other constituents disrupt the regular ordering of fibrils. Our data suggest that interactions of cellulose with *fah1*-*2* and *C4H::F5H fah1*-*2* provide somewhat less disruption of cellulose order than the interactions in wild type, while the degree of crystallinity remains unchanged (Fig. 2). Aldehyde containing lignins have lower coherence length, suggesting potentially greater interactions, again without altering crystallinity. *ref3*-*2*, with lower lignin content, might be expected to place fewer constraints on the organization than wild type, but lowered crystallinity could offset that, perhaps leading to the observed lower coherence length. For *ccr*, the balance between lowered crystallinity and lowered constraints results in no change in observed coherence length.

In principle, we could also calculate the coherence length for the unoriented fraction of cellulose using curves similar to those in Fig. 13. In practice, the (0 0 4) reflections in these traces are weak and broad, making accurate measurement of coherence length difficult, while clearly indicating that the coherence length is significantly less for the unoriented fraction than for the oriented fraction of cellulose. In all likelihood, this reflects the greater curvature of fibrils expected in the unoriented fraction.

### Packing of cellulose fibrils

**Fig. 8:**
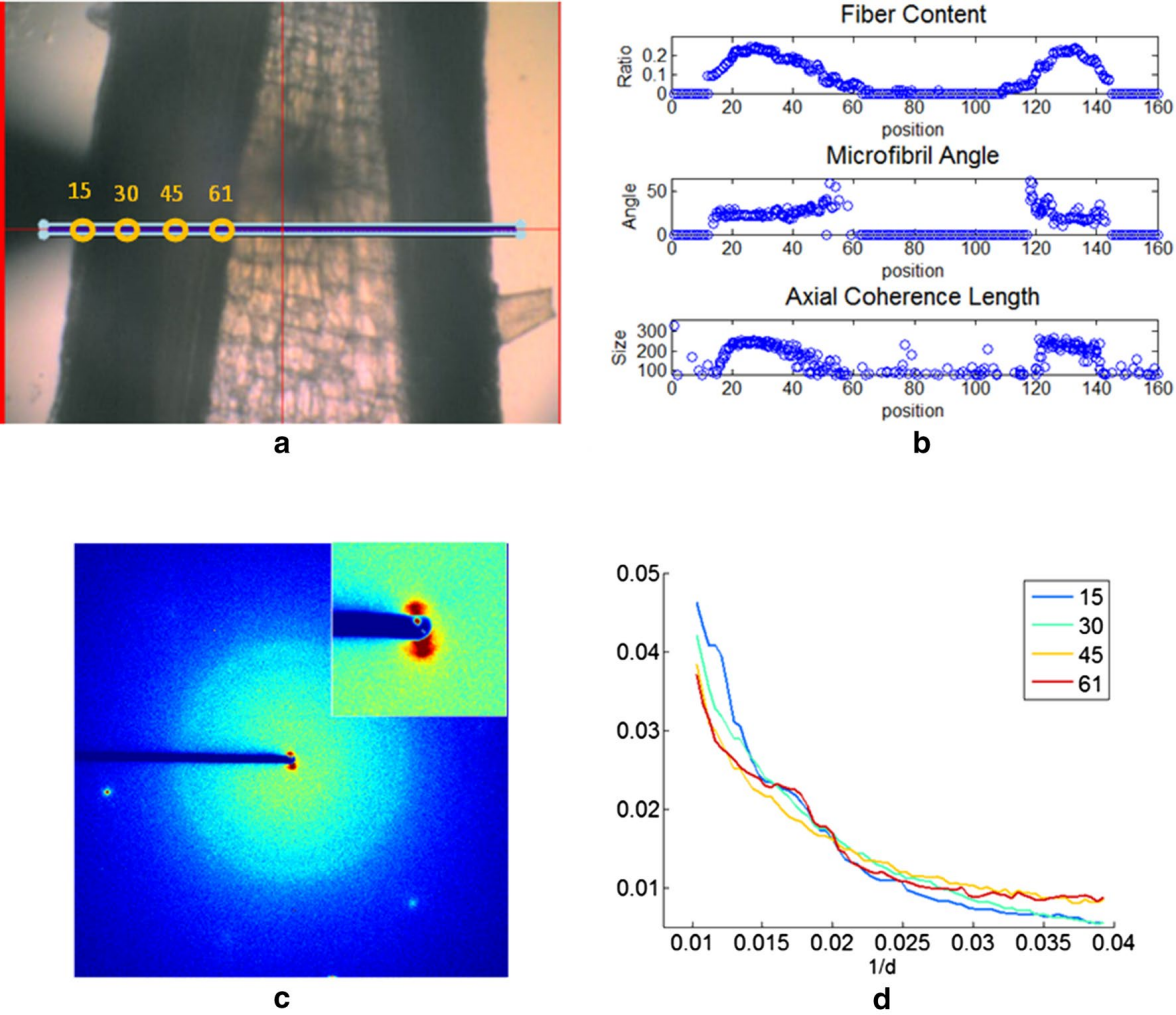
Modulation of small angle scattering by interference due to packing of cellulose fibrils was observed to be strongest in the region near the pith. **a** Optical image of stem, the grid containing 3 × 160 points corresponds the position of the microdiffraction scan. **b** The distribution of fiber content and microfibril angle across the stem. **c** Diffraction pattern collected at position 61 close to the pith exhibits a strong modulation of small angle scattering (*inset*) attributed to partially ordered packing of cellulose fibrils. **d** Comparison of intensity at small angle region for four positions from epidermis to pith, and exhibiting the modulation at ~0.017 Å^−1^ due to interference

The small angle region of the diffraction patterns collected here provides structural information about features ranging from 25 to 100 Å in size. The intensity distribution in this region corresponds to the scattering from individual cellulose fibrils. When the fibrils are arranged in an organized fashion, regularly spaced side-to-side, the intensity is modulated by an ‘interference function’ that provides information on the spacing of fibrils in the material [7, 8, 27, 28]. Figure 8 shows the intensity distribution in the small angle region of a wild-type sample, an enlargement of the small angle region of exposure 61 and equatorial traces for exposures 15, 30, 45, and 61. The trace through the exposure (taken from a diffraction pattern of a region immediately adjacent to the pith) shows a modulation of the small angle scattering intensity with peak at 1/day ~0.017 Å^−1^, suggesting that the fibrils are spaced with a nearest neighbor distance of approximately 60 Å. The observation of this interference near the pith is unexpected because this is the region of the stem with the lowest (observable) oriented fibril content. If the region was homogeneous, the spacing observed would imply an oriented fibril content of at least 25 %, far higher than we observe. Therefore, the region must be highly heterogeneous, with the small fraction of oriented fibrils well—ordered in spatially confined regions.

### Impact of water storage on molecular structure of plant cell wall

In addition to collecting SXMD data on samples harvested 3 days prior to data collection, we collected SXMD data on samples that were stored in water for 30 days at room temperature prior to data collection. Our observations show that water storage contributes to observable structural variations in a lignin-dependent manner.

### Impact on oriented fiber content

**Fig. 9:**
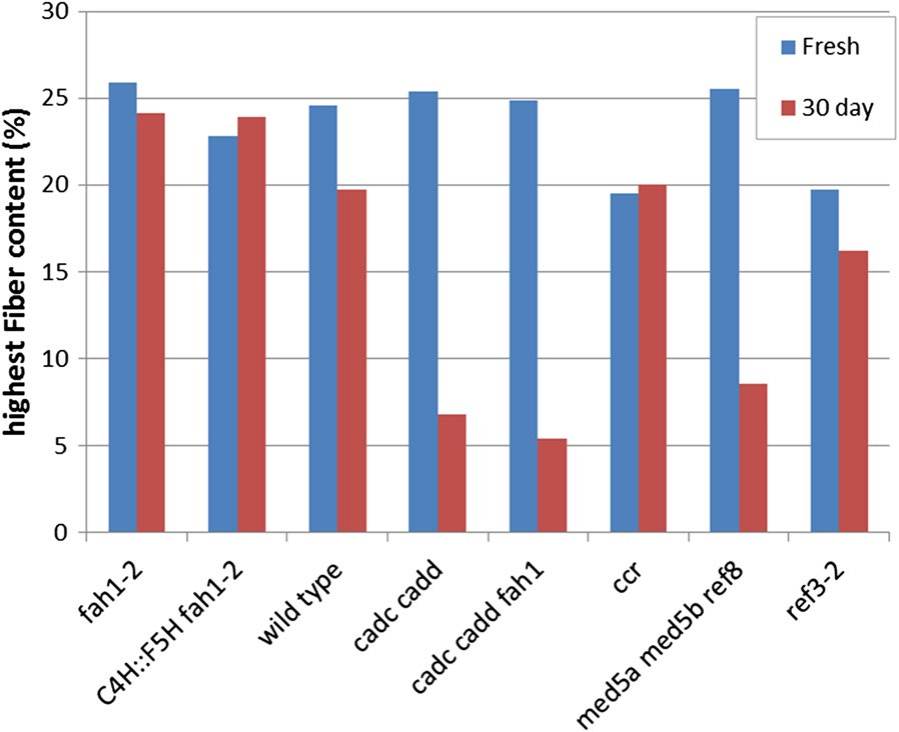
Comparison of the highest oriented fiber content observed in each sample. The *blue bars* correspond to the samples dried 3 days after harvest. The *red bar* corresponds to the samples dried after storage in water for 30 days. Experiments were replicated using two sets of samples grown, harvested, and analyzed months apart and reproducing all the trends represented here

Storage reduced the observed fiber content of wild type only slightly and the reduction of fiber content in *C4H::F5H fah1*-*2* and *fah1*-*2* was negligible. However, storage significantly lowered the levels of crystalline cellulose in the high aldehyde samples, *cadc cadd*, *cadc cadd fah1,* and *med5a med5b ref8* sample even though their fiber content was comparable to wild type in fresh samples (shown by red bar in Fig. 9). These results were replicated on two sets of samples grown, harvested and analyzed independently at different times. These observations suggest that lignin organization may be important for protecting cellulose from degradation that may occur in aqueous environments over time. Other samples exhibited less change on storage. Interestingly, Fig. 9 shows that, although *ccr* samples stored in water for 3 days exhibit lower fiber content than wild type, *ccr* does not show a decrease in oriented fiber content after storage. No variant exhibited a significant increase in oriented fibril content over wild type.

### Impact on axial coherence length

**Fig. 10:**
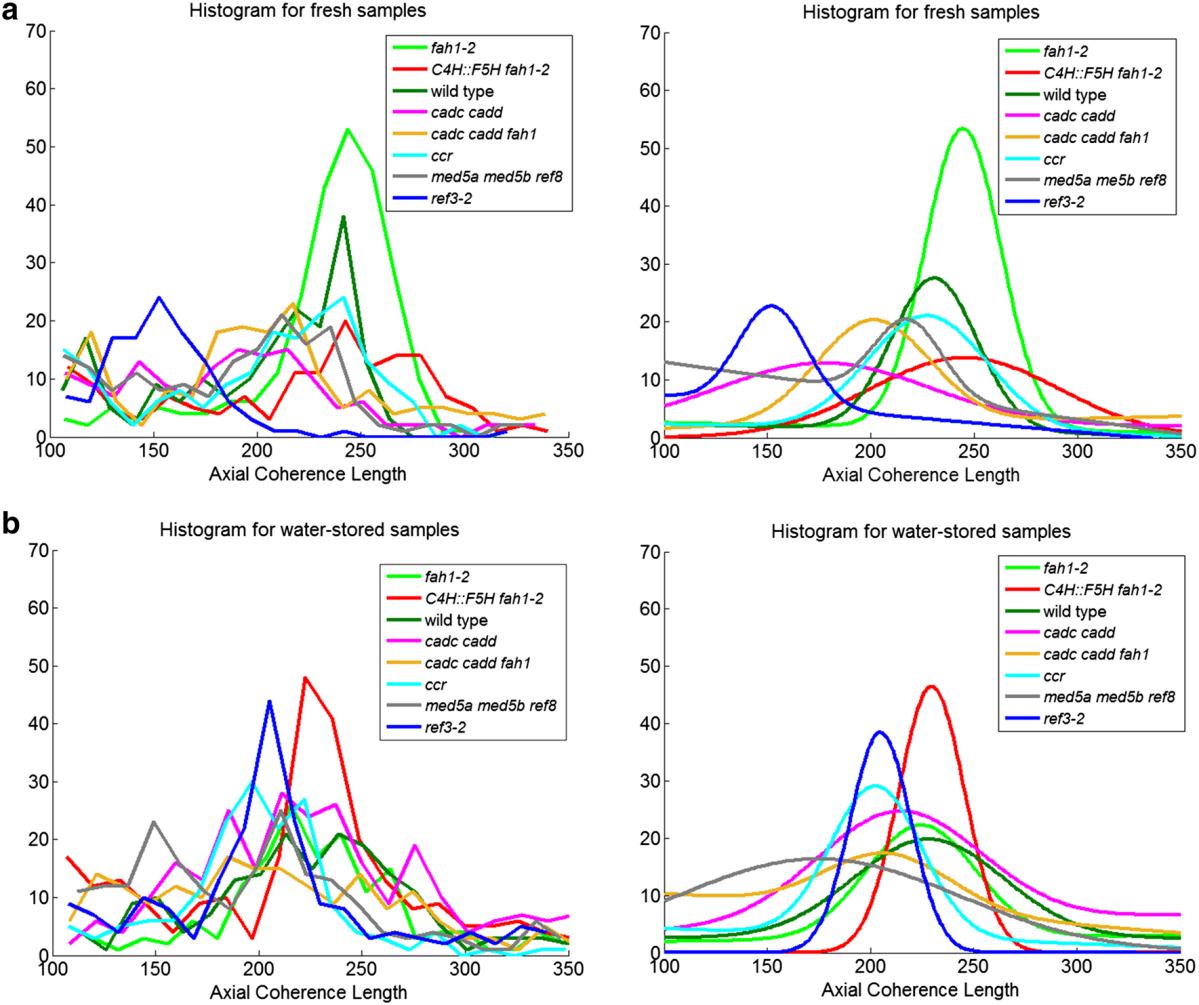
**a** Histogram of axial coherence length for samples dried fresh and after 30 days in water. The axial coherence length differs across the stem, tending to be the largest in the vascular tissues. **b** Gaussian curves fit to each of the histograms in **a** suppress the effect of random variations and facilitates visual comparisons among the samples

Figure 10 shows that exposure to water for 30 days results in most cases in a decrease in coherence length. *ref3*-*2* appears to be the exception. It's very low coherence length appears to increase on storage in water—perhaps through the relaxation of cross-linking constraints on its cellulosic structures. Interestingly, there is no direct correlation between the coherence length and fiber content for the lignin mutants studied here. This may be the result of two competing factors—cellulose crystallinity, which should correlate with increased coherence length; and cross-links to other constituents which should correlate with decreased coherence length.

## Discussion

A complex interdependency of cellulose and lignin assembly into the plant cell wall seems essential to production of the intricate nanoscale architecture required for cell wall integrity, strength, and resiliency. Nevertheless, a clear demonstration of this interdependency is complicated by the possibility of indirect effects and individual variations. By querying all stem tissues and multiple individual plants with ~16,000 diffraction patterns, we sought to minimize the potential impact of individual variation and tissue-specific effects. The systematic and reproducible differences we have observed in cellulose structure among *Arabidopsis* variants with altered lignin metabolism provide compelling evidence that lignin contributes to the organization of cellulose in the plant cell wall and that the specifics of lignin composition impact resilience to degradation.

The vegetative apparatus of *Arabidopsis* is a ground rosette that develops a lignified flowering stem [29]. Besides the xylem vessels, which are lignified, the interfascicular parenchyma of the flowering stem differentiates into highly lignified fibers with growth. Under certain growth conditions, lignin in *Arabidopsis* can represent up to 18 % of the dry weight of the extractive-free mature stem and contain both S and G units [29]. Alteration of the lignin composition and content of the stem in some cases leads to significant changes in phenotype. For instance, fah1-2 blocks F5H causing a deposition of primarily G-lignin [13, 14] an F5H overexpressing transgenic plant deposits mostly S-lignin [15, 16]; *ccr* and *ref3*-*2* lead to deficient lignin deposition [19–21]; and a triple mutant *med5a med5b ref8* contains predominantly H-lignin [6]. These phenotypic alterations are obvious in the whole plant and at the level of electron microscopy which reveals in some variants wholesale disruption of the cell wall structure and collapse of the vascular tissues. Finer scale disruptions have been largely unexplored. How does the disruption of normal lignin synthesis lead to the molecular changes that enforce these phenotypic changes? By using scanning X-ray microdiffraction of lignin variants, we sought to reveal the changes in cellulose architecture triggered by alterations in lignin composition.

### Mutations in the lignin biosynthetic pathway affect deposition of cellulose

The largest fiber content in the *Arabidopsis* stem is in the vascular tissue. We observed that variants with *fah1*-*2*, *med5a med5b ref8*, and aldehyde [*cadc cadd* and *cadc cadd fah1*] exhibit maximum fiber content comparable to that of wild type. In plants with abnormally *C4H::F5H fah1*-*2* lignin, the maximum fiber content is modestly reduced. Sun et al. [30] reported that the syringyl to guaiacyl (S/G) ratio will cause the changes in carbon hydrate composition, like cellulose. This may lead to modest fiber content variation. However, a significant decrease of fiber content is observed in the *ccr* and *ref3*-*2* mutants (Fig. 2) that also have decreased lignin content. Turner et al. [2] and Goujon et al. [19] reported that the *ccr* mutants have a deficient of cellulose deposition in the stem, consistent with our results. Similarly, the *ref3*-*2* variant affects both lower lignin and cellulose deposition in the secondary cell wall [21], as reflected here. These observations indicate that lignin is essential to the deposition of cellulose fibril within the plant cell wall. The subnormal deposition of cellulose fibrils in *ccr* and *ref3*-*2* may be a factor in the observed dwarfism of these plants.

### Alteration in lignin composition accelerates the degradation of order during storage

Although the lignin contents of *cadc cadd*, *cadc cadd fah1, and med5a med5b ref8* mutants are reduced, the highest observed fiber content of these mutants is similar to wild type. Nevertheless, after 30 days in water, thin sections of these plants exhibit significantly lowered content of oriented cellulose fibrils, indicating that the lowered lignin content has increased their susceptibility to degradation processes. In contrast, plants with *fah1*-*2* or *C4H::F5H fah1*-*2* show no significant alteration in fibril content after 30 days in water. This indicates that lignin exhibiting high levels of G or S subunits may confer some protection of cellulose fibrils from the relevant degradative processes.

### Altered lignin content changes the orientation of cellulose fibrils

Lichtenegger et al. [10, 11] and Riekel et al. [12] observed the variation of microfibril angle by using micro X-ray technology. The microfibril angle is thought to reflect the helical arrangement of cellulose microfibrils around the plant cell. How the helical geometry of the architecture of the plant cell determines the microfibril angle had been discussed by Emons and Mulder [23] and Lichtenegger et al. [10, 11]. The microfibril angle represents the tilt of cellulose fibril relative to long axis of the stem. The average tilt of cellulose fibrils in the vascular tissues is never more than ~30° in these variants. For comparison, the maximum tilt of cellulose microfibrils in roots of *Arabidopsis* was reported to be ~45° [31, 32].

Since deposition of lignin within the plant cell wall decreases as one approaches the pith [2], Liu et al. [9] noted that the reduction of lignin content in the cell wall correlates with a decrease in fiber content which inversely correlates with changes of microfibril angle in *Arabidopsis* stem. We repeated those observations here, but noted that this pattern is not replicated in all variants. The correlation in wild type suggests that lignin may be essential for anchoring cellulose microfibrils within the polysaccharide matrix at appropriate tilts relative to the stem axis. Disruption of lignin synthesis may lower the resilience intrinsic to these structures.

Although orientation of cellulose is sufficient for measurement of fibril reflections in wild-type plants and those that deposit primarily S-lignin, in several of the mutants, the degree of orientation was inadequate for estimation of fibrillar angle. This is especially true for mutants with severe reduction of lignin content. Interestingly, where measurable, the mean value of microfibril angle in these mutants is considerably larger than others. We conclude that lowered lignin content leads to a considerable disruption in the orientation of cellulose fibrils in a manner that mirrors the lower degree of orientation of cellulose in the pith.

### The regularity of cellulose fibrils varies with lignin mutants

Coherence length is an important feature reflecting the integrity of cellulose fibrils within plant cell walls. The most obvious reduction in coherence length is observed in *ref3*-*2*, a plant with severe lignin deficit [21]. It is possible that the lowered coherence length may be due to curvature of the fibrils. These observations support the necessity of lignin for maintaining proper assembly of cellulose fibrils within cell walls. The loss of integrity of the cellulose fibrils may be an underlying contributor to the extreme dwarfism displayed by this plant. The implication is that lowered lignin content disrupts the natural order of cellulose fibrils within the cell wall. When this happens, it is possible that the cellulose is deposited in the cell wall in such a way that it is susceptible to cell wall enzymes normally involved in wall restructuring. The implication is that cellulose may be produced at normal levels, but with the vast majority of cellulose deposited in an aberrant fashion, most of it is digested and recycled, resulting in a dramatic stunting of plant growth.

The *C4H::F5H fah1*-*2* and G plants exhibit very similar coherence lengths to the wild type. The aldehyde mutations show lower coherence length than wild type but greater than *ref3*-*2*. Nevertheless, water storage caused significant fiber content reduction as well as much weaker (0 0 4) reflections. Although coherence lengths of *cadc cadd* and *cadc cadd fah1* do not change as much as *ref3*-*2* for water-stored samples, weakness of axial reflections from these mutants indicates that cellulose fibrils are probably highly curved. This may alter the physical properties of the cell walls, lowering stiffness of cell wall and leading to limp floral stem. We noticed that *med5a med5b ref8* is the only variant that exhibits decreased fiber content and coherence length. Therefore, water storage may lead to a digestion of cellulose as well as increased cellulose fibril disorder.

Langan et al. [33] reported that reduction of lignin induced larger elementary fibrils of diameter of 60–70 Å by molecular dynamic simulation. The equatorial scattering reported here is not consistent with the coalescence of fibrils and presumably reflects a distinct phenomenon. Coalescence would result in sharper reflections, whereas we see broadening of the equatorial reflections in the samples with lignin deficit (Fig. 3).

### Molecular architecture in the stem of lignin mutants

**Fig. 11:**
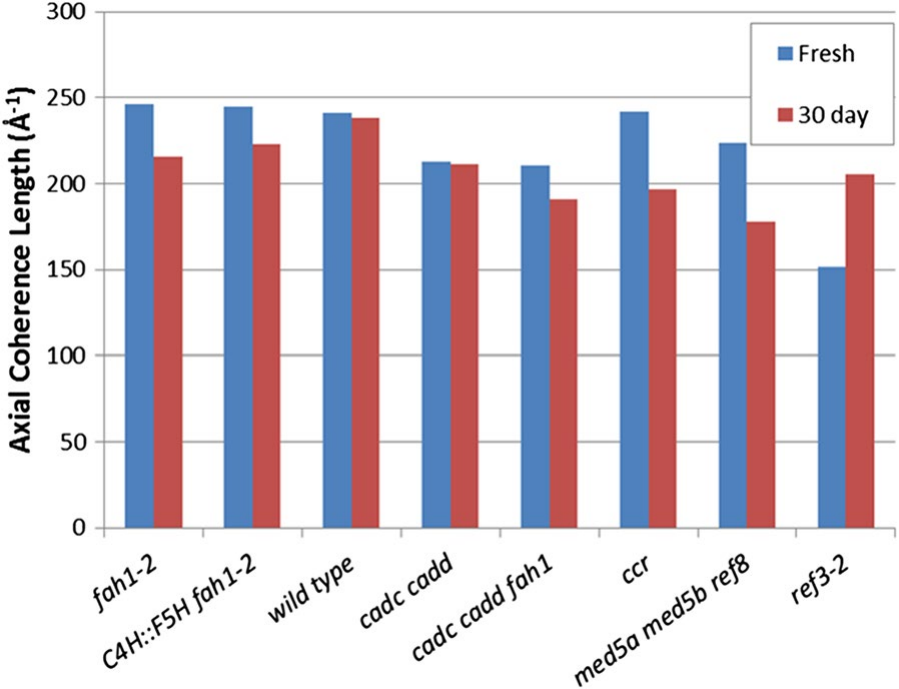
Comparison of maximum coherence lengths for each of the 16 samples. This is a compilation of the peak positions of the smoothed curves shown in Fig. 10a, b

The oriented fiber content, microfibril angle, and axial coherence lengths are presented for 16 samples in the Additional file 1. Those charts provide a comprehensive overview of the tissue-specific variations in these properties among the samples. Individual variations are inevitable in comparisons of these types, but replicate experiments indicated that the overall trends reported here are representative. No sample exhibited significantly higher oriented fiber content than wild type. Disruption of lignin biosynthesis resulted in either a decrease in the proportion of oriented cellulose fibrils, or no apparent change. Plants with *fah1*-*2* or *C4H::F5H fah1*-*2* behaved quite similar to wild type, with *C4H::F5H fah1*-*2* having, perhaps, somewhat lower oriented cellulose fiber content. Interestingly, they both displayed average axial coherence lengths comparable or slightly higher than that of wild type. Ciesielski et al. [4] reported that wild type and *fah1*-*2* exhibit only very subtle ultrastructure changes even after maleic acid treatment. This is consistent with our observation that the highest fiber content and axial coherence length of *fah1*-*2* are slightly larger than wild type and other samples. Figures 9 and 11 indicate that water storage has little impact on cellulose fibrils within plant cell wall of *fah1*-2. However, the structural alteration of *C4H::F5H fah1*-*2* due to the water storage is not as great as observed for maleic acid treatment. For *C4H::F5H fah1*-*2,* catalytic conditions, such as thermal or ionic catalysis, are essential for improvement of degradation of cell wall.

*cadc cadd*, *cadc cadd fah1,* and *med5a med5b ref8* plants also had similar fiber content to wild type, but displayed significant sensitivity to degradation during storage in water. They exhibit a broader distribution of axial coherence lengths than wild type, suggesting inhomogeneity in the spatial constraints on cellulose organization. *ccr* and *ref3*-*2* had lower oriented fiber content than wild type but exhibited little change in oriented fiber content in reaction to storage. However, their axial coherence lengths became more homogeneous after storage, with that of *ref3*-*2* increasing significantly. This suggests that the spatial constraints on cellulosic structures decreased in these plants during storage. Transmission electron microscopy (TEM) shows that secondary cell walls are much more disorganized for *cadc cadd*, *cadc cadd fah1*, and *med5a med5b ref8* compared to *fah1*-*2*, *C4H::F5H fah1*-*2*, and wild type, respectively [3, 6]. Their digestibility increase may be due to the observed swelling, allowing higher penetration of reagents into the of plant cell wall for digestive reactions.

## Conclusions

### Tissue-specific variation in cellulose order

SXMD demonstrated that in wild type, the apparent cellulose fiber content varies across the stem with highest level near the middle of the vascular tissues. The microfibril angle is relatively constant through the vascular tissue but increases toward the pith to 35° or more before becoming unmeasurable due to complete disorientation and low abundance of cellulose within the pith. The observation of low-microfibril angle in vascular tissue is consistent with cellulose orientation observed by Confocal Raman microscopy and VCA [24, 25]. Introducing chemical component analysis, like Raman spectroscopy (Sun et al. [30]) and NMR, combining SXMD results, could provide deeper understanding of the impact of variation of chemical composition on molecular architecture within plant cell wall. The axial coherence length is relatively constant across the vascular tissues of wild-type plants, but decreases significantly toward the pith (probably due to curvature of the cellulose fibrils which may contribute to the complete lack of orientation of cellulose fibrils within the pith). After 30 days in water storage, maximum fiber content of wild type decreases about 20 % and microfibril angle becomes far less constant. Axial coherence length also becomes somewhat less constant, but, on average, is not too different from what is observed in fresh tissue.

### Variants

Plants with *fah1*-*2* and *C4H::F5H fah1*-*2* lignins do not appear significantly different from wild type in terms of the organization and orientation of cellulose in the stem. *ccr* and *ref3*-*2* are the only variants that show significant decrease in cellulose fiber content compared to wild type. Both of them show a roughly 20 % drop. *ref3*-*2* exhibits lower lignin content than wild type with almost complete collapse of vascular elements [21]. Given the collapse of vascular elements, it is perhaps surprising that the cellulose fiber content remains as high as it does. The coherence length of fibrils in *ref3*-*2* is also significantly smaller than wild type, again indicative of significant disruption of the order typical of vasculature in wild type plants. The lower cellulose fiber content in *ccr* may be due to different interactions between cellulose and the ferulic acid-containing lignin that is abundant in *ccr* during deposition of cellulose. Unlike *ref3*-*2*, the coherence length of cellulose fibrils in *ccr* plants is only slightly lower than that in wild type, indicating somewhat different levels of restraints on the cellulose in these plants.

### Storage in water

Storage in water for 30 days has a dramatic impact on the cellulose organization of some plants. Apparent cellulose fiber content decreases by ~20 % in wild-type plants. *fah1*-*2* and *C4H::F5H fah1*-*2* plants appearing resilient to structural changes during storage in water and little difference between fresh and stored samples were observed. However, in aldehyde-rich or *med5a med5b ref8* plants, the decrease is closer to 80 %. This observation suggests that lignin has a significant role to play in protecting these tissues from degradation and that change in the nature of the lignin can dramatically alter the speed with which these tissues degrade. Lignin content, per se, is not the overriding factor, as *ref3*-*2*, with significantly lowered lignin content, appears far more resistant to structural degradation during storage than the high aldehyde or *med5a med5b ref8* plants. *med5a med5b ref8* and *ccr* decrease by ~15 % and, counterintuitively, the coherence length observed in *ref3*-*2* appears to increase on storage in water. One possible explanation is that storage leads to breakage of some of the cross-links between cellulose and other cell wall constituents and this frees up cellulose fibers to take on a lower energy configuration in which the crystallinity of the remaining fibrils is increased (even though the overall fiber content has decreased).

## Methods

### Separation of oriented and disoriented scattering

**Fig. 12:**
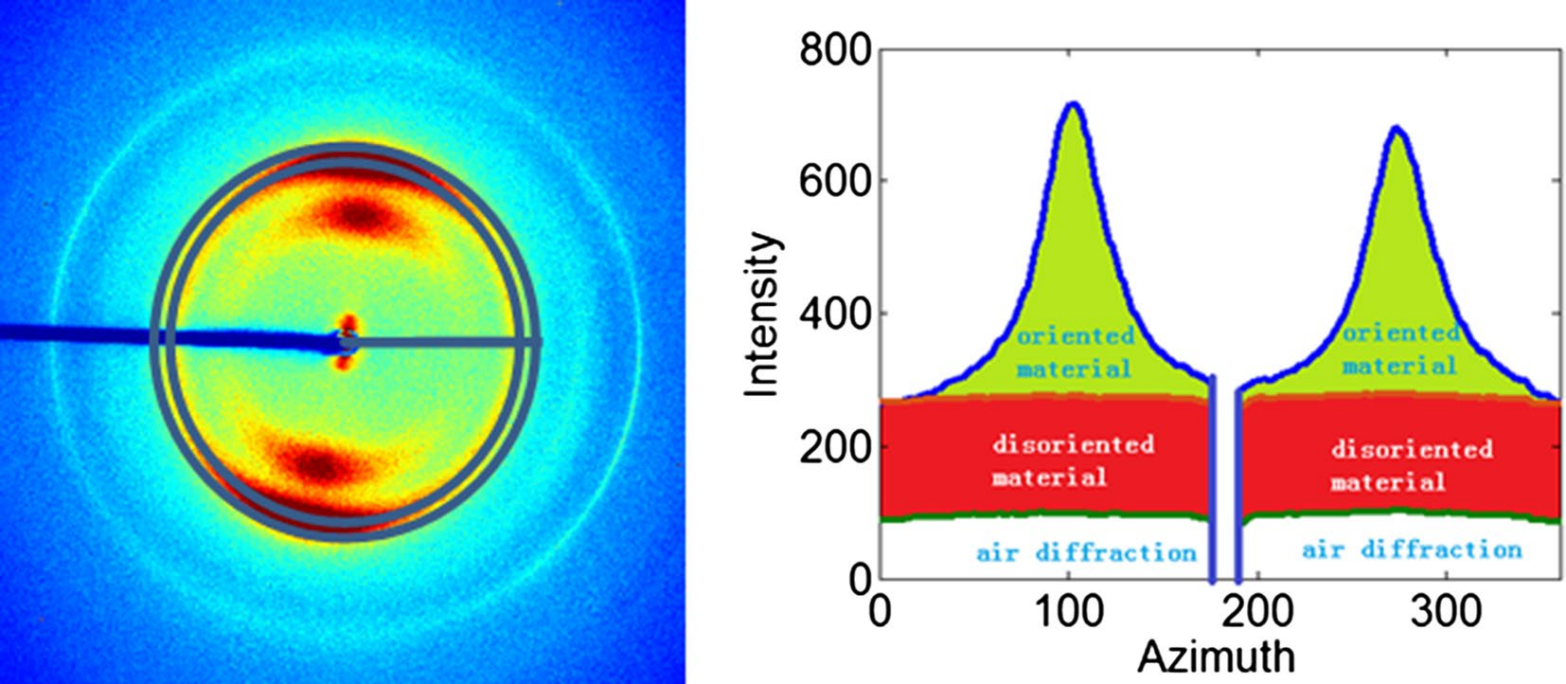
The scattered intensity was separated into circularly symmetric (*red* and *white* in diagram to* right*) and oriented fractions (*green in right*) using intensity at the scattering angle with greatest intensity (the position of the (2 0 0) reflection marked as a circle in the scattering pattern to the *left*)

**Fig. 13:**
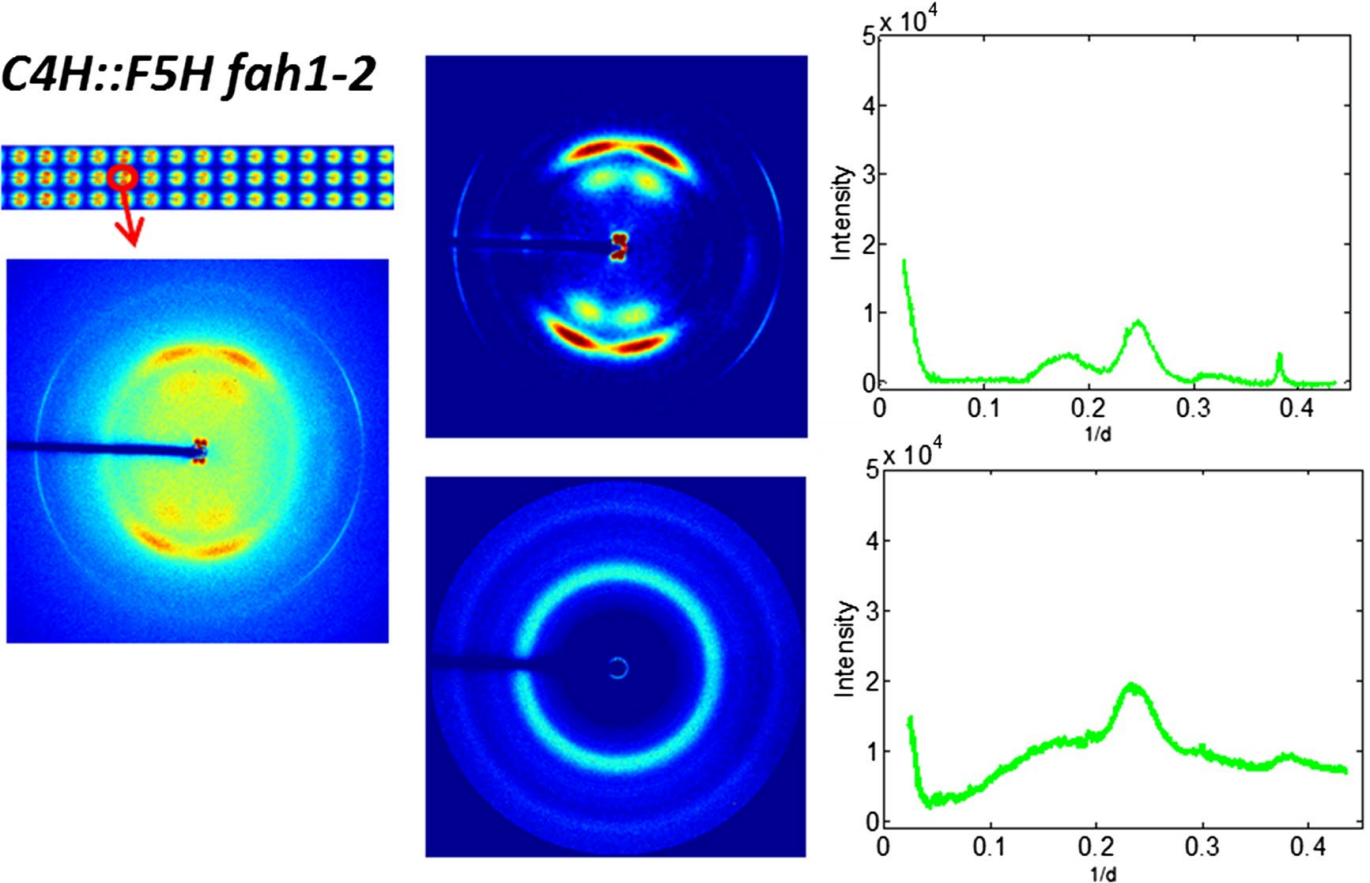
Example of separation of circularly symmetric and oriented intensities. Each pattern from the *C4H::F5H fah1*-*2* sample was analyzed and separated into oriented (*top*, *middle*) and unoriented (*bottom*, *middle*) fractions. The intensities were then plotted as a function of spacing 1/day (roughly proportional to scattering angle) for the oriented (*top right*) and unoriented (*bottom right*) fractions. The oriented portion exhibited well defined (1 1 0)/(1 −1 0), (2 0 0), and (0 0 4) reflections characteristic of scattering from cellulose Iβ. The unoriented fraction had an intensity distribution rather different from that expected for cellulose and probably represents a sum of scattering from all constituents of the tissue except that from the oriented cellulose fibrils

Diffraction patterns displayed a wide diversity in the degree of orientation with many patterns, such as those from the pith, displaying essentially no oriented scattering and patterns from the xylem of some plants exhibiting significant orientation. Scattering from cellulose fibrillar structure contributes to the majority of oriented diffraction. Studies of cellulose by X-ray often utilize crystalline index (CI) as a quantitative metric of the degree of crystallinity of cellulose fibrils. Several measures similar to CI appear in the literature [34, 35]. Park et al. [35] indicate that CI can be determined by comparing the intensity maximum and minimum in the range of 0.1–0.3 Å^−1^ or through fitting of cellulose reflections with multiple Gaussian functions. Fernandes et al. [34] postulate an asymmetric function for CI calculations in order to explain their observations. Although these different empirical measures lead to somewhat different values and reflect somewhat different properties, most exhibit similar trends for similar tissues. In order to enable comparison of cellulosic organization in different samples, we introduce ‘fiber content’ as an alternative to CI utilizes a larger fraction of the observed data and whereby represents a more accurate measure of the relative degree of cellulose crystallinity in different regions of each sample. We separate scattering into oriented and disoriented components as described in “Methods” section. Briefly, as shown in Fig. 12, the separation was made by isolating the anisotropic part of the pattern (green on the right) from the isotropic part (red and white on the right). The scattering from air was accounted for by subtraction of scattering from the camera in the absence of a sample. The separation of oriented from disoriented scattering is carried out by fitting of the intensities with a Gaussian plus a constant. The intensity as a function of scattering angle could then be calculated for both of these fractions resulting in the intensity distributions in Fig. 13. The intensity observed for the oriented part of the pattern exhibits most of the features expected in scattering from cellulose, including the (1 1 0)/(1 −1 0), (2 0 0), and (0 0 4) reflections. Scattering in the disoriented part of the pattern exhibits an intensity distribution rather different from that observed in the oriented part of the pattern, with broader peaks and substantial increase in features not normally associated with scattering from cellulose. The impression is that the unoriented material is a combination of scattering from poorly ordered cellulose and a preponderance of noncellulosic materials.

### Sample preparation

Wild-type and lignin biosynthetic mutants of *Arabidopsis* were grown for 6 weeks under long day condition (16 h light/8 h dark), and the bottom of primary stems were cut into 100-µm-thick longitudinal sections. One sample set was immersed in water at room temperature for 3 days and subsequently dehydrated in air at room temperature for 24 h before data collection. A second set was immersed in water at room temperature for 30 days prior to being dehydrated.

### Scanning X-ray microdiffraction

X-ray diffraction data were collected at beamline 23ID-B at the APS using the scanning microdiffraction capability developed for use in macromolecular crystallography [36]. Samples were aligned using a coaxial optical microscope with a hole down the optical axis to accommodate the X-ray beam thereby allowing precision alignment of the beam to select positions on the sample. A 5-μm beam size was used and samples were stepped along a 5-μm grid with a wide-angle diffraction pattern collected at each grid point. A specimen-to-detector distance of 300 mm was used with an X-ray wavelength 1.033 Å (X-ray energy of 12 keV). The exposure time was 2 s. Patterns were recorded with a MAR300 detector with 2048 × 2048 pixels in an area of 30.00 cm × 30.00 cm. Pixel size was 146 micron. Reciprocal spacing 1/day = 2sin(*θ*)/*λ*, *θ* is the Bragg angle, and *λ* is the wave length.

Background scattering was estimated from the first and last diffraction patterns of each row of the montage. These patterns were collected at positions outside of the sample and constitute diffraction from air and residual scatter from camera elements within the experimental environment. These backgrounds were subtracted from all diffraction patterns prior to all other data analysis.

### Estimation of microfibril angle

Scattering from fibers within real tissue may overlap at micron-scale due to structural arrangement and sample preparation. Lichtenegger et al. [9, 10] and Liu et al. [11] reported that the fiber orientation of cellulose could be determined by simple analyses of diffraction patterns. Therefore, we developed an algorithm to determine and separate the fiber orientation by azimuthal intensity distribution of diffraction patterns.

Microfibril angles were estimated from the angular variation of intensities in the polar coordinate system (Fig. 12). Azimuthal positions of peaks were determined by fitting of Gaussians to the intensity as a function of *θ* at a radius corresponding to the (2 0 0) reflection of the cellulose Iβ structure.

### Separation of oriented material and disoriented material

Each diffraction pattern was separated into oriented and disoriented fractions (see Fig. 12). The unoriented fraction was determined from the isotropic distribution at each scattering angle (1/day-spacing). Air scattering was derived from patterns at the start and end of each row of the montage which were chosen to be outside the spatial extent of the sample. For each 1/day-spacing, subtraction of disoriented material and air scattering resulted in a distribution exhibiting one or more peaks that could be approximated by Gaussian distributions.

### Calculation of fiber content

**Fig. 14:**
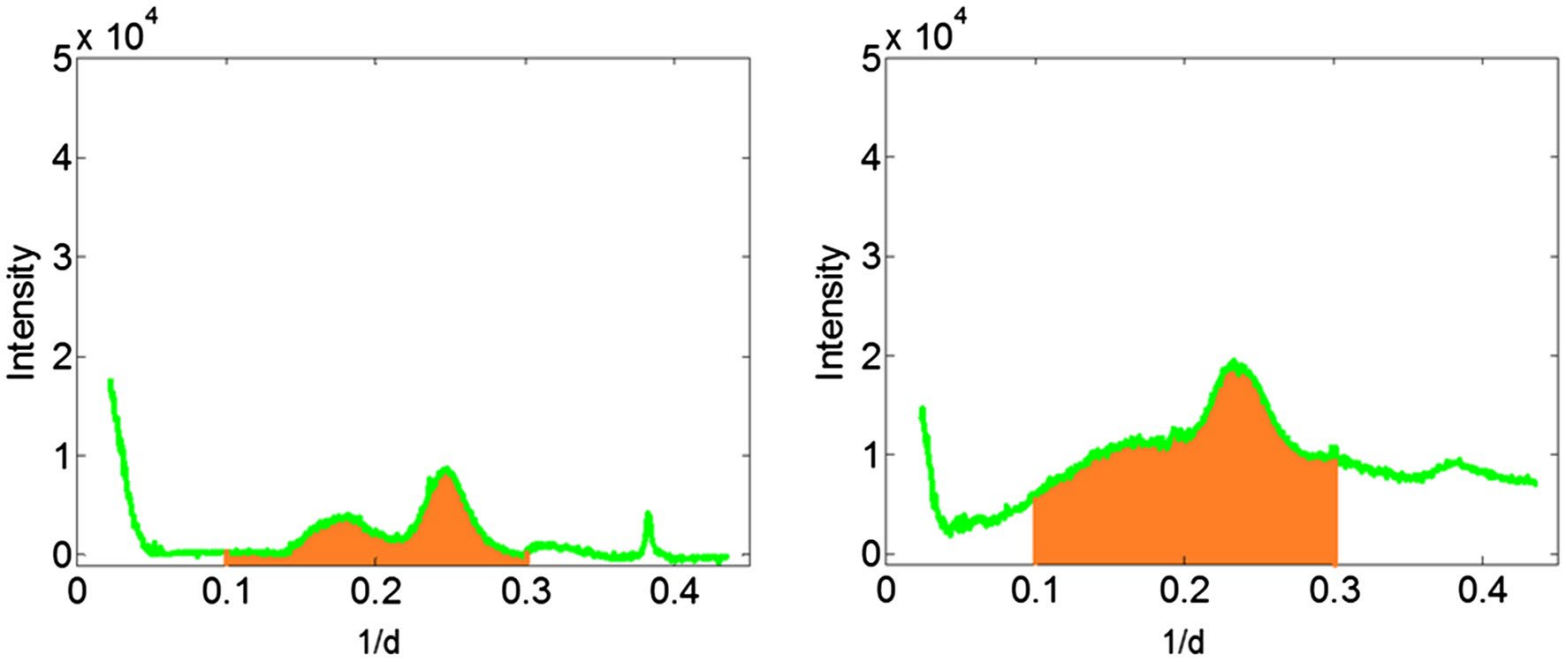
Calculation of oriented fiber content. Oriented and circularly symmetric intensities are separated as demonstrated in Fig. 12. The intensity of oriented material (*left*) and unoriented material (*right*) are then integrated over a range of spacing corresponding to the positions of the (1 −1 0) and (2 0 0) reflections from cellulose (indicated in *orange* in the figures and spanning 0.1 < 1/day < 0.3 Å^−1^). The proportion of oriented fibrillar material is then calculated as indicated in the text

Fiber content was calculated to exhibit the oriented fibril content. Calculation is a linear combination of the intensities of oriented material and disoriented material, as shown in Fig. 14, the calculation was

$$I_{{{\text{fiber}}\;{\text{content}}}} = \sum {I_{{{\text{oriented}}\;{\text{material}}}} /\left( {\sum {I_{\text{oriented}} } + \sum {I_{\text{disoriented}} } } \right)}$$

$I_{{{\text{fiber }}\;{\text{content}}}}$ is a measure of oriented fibril content, $I_{{{\text{oriented}}\;{\text{material}}}}$ is the normalized integral intensity of elementary fibrils, and $I_{\text{disoriented}}$ is the normalized integral intensity of amorphous component.

### Calculation of axial coherence length

A cellulose fibril is nearly crystalline, but because of its extreme length, very large surface area, curvature and tendency to twist, the crystallinity is imperfect. Because of local stretching, twisting, and curvature, the periodicity of a cellulose fibril varies along its length. This leads to a phase difference in the molecular repeating structure that increases progressively along the length of the fibril. The coherence length is the distance along the fibril beyond which there is no ordered phase relationship. Therefore, the coherence length may provide insight into the interactions of cellulose fibrils with other cell wall constituents, and a change in coherence length may reflect a disruption in those interactions, a perturbation in the ordered process by which cellulose fibrils are assembled into the cell wall or enhanced distortion of the cellulose fibrils caused by increased physical constraints due to interactions with other cell wall constituents.

**Fig. 15:**
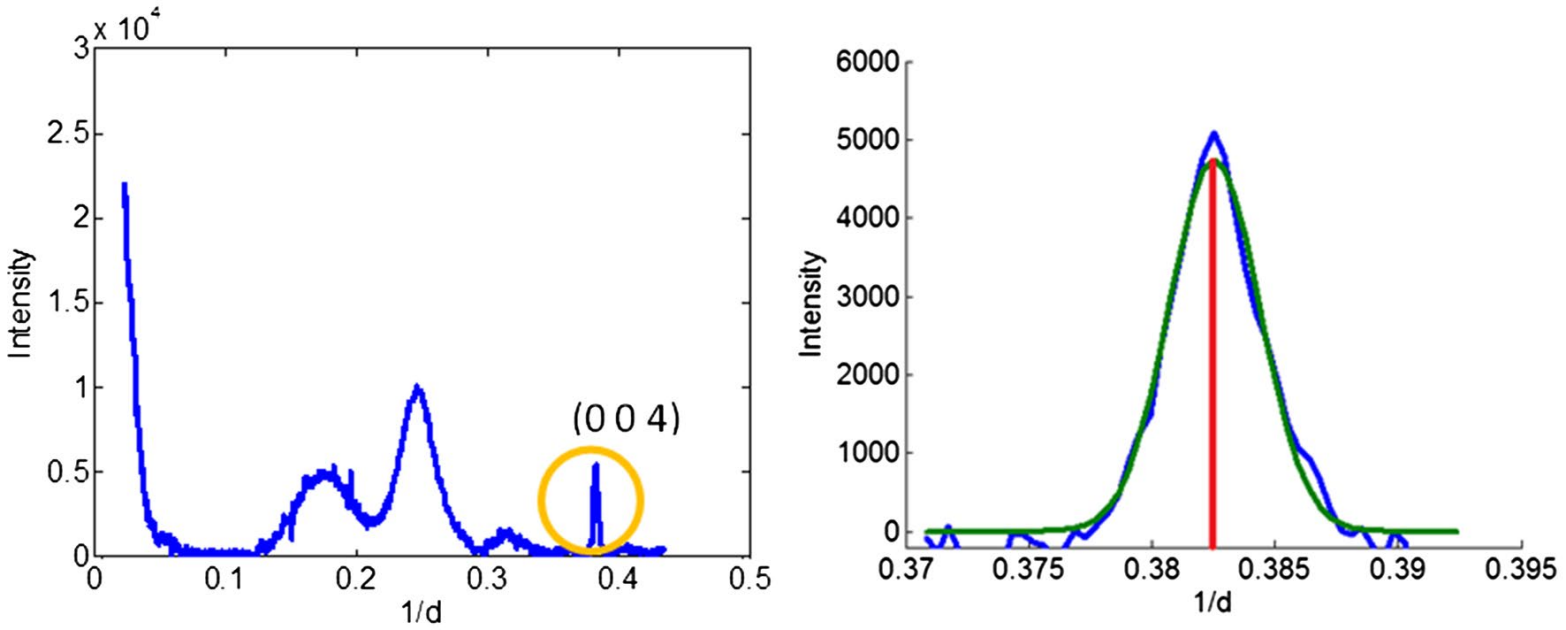
The calculation of axial coherent length for (0 0 4) reflection. The* Left image* is a trace of the oriented intensity including the (0 0 4) reflection of cellulose fibrils. An enlargement of the (0 0 4) on the* right* shows the background subtracted reflection and (*blue curve*) and a Gaussian function (*green curve*) fit to intensity distribution. *Red line* indicates the maximum position of Gaussian curve

Axial coherence length was estimated from the breadth of the (0 0 4) reflections calculated from the Scherrer equation. Figure 15 shows the Gaussian fitting of (0 0 4) reflection of cellulose fibrils. Then coherent length could be calculated as follows:

$${\text{Coherence}}\;{\text{length}} = \frac{K\lambda }{\beta\,\cos \left( \theta \right)}.$$

The green curve shows a Gaussian function fitting the reflection. The *θ* can be determined by the peak position of the Gaussian and *β* is the half width of Gaussian function. *K* is constant 0.9. *λ* is wavelength of incident X-ray.

## Additional file

10.1186/s12014-016-9116-y Variations of oriented fiber, microfibril angle, and axial coherence length across the stem for each mutant and wild type have been exhibited in figures S1–S8 of supplement material.

## Abbreviations

SXMD: scanning X-ray microdiffraction
*fah1*-*2*: a ferulic acid hydroxylase 1 (f*ah1*-*2*) mutants
*C4H::F5H fah1*-*2*: a *F5H* overexpressing transgenic plant
*med5a med5 ref8*: a triple mutant *med5a med5b ref8*
*cadc cadd*: a plant-harboring lesions in both *cadc* and *cad*d
*cadc cadd fah1*-*2*: a triple mutant, *cadc cadd fah1*
*ccr*: a plant with down-regulation of cinnamoyl CoA reductase 1
*ref3*-*2*: a *ref3*-*2* mutant
