# Supplementary material for: The impact of alterations in lignin deposition on cellulose organization of the plant cell wall
Source: Biotechnol Biofuels. 2016 Jun 17;9:126. doi: 10.1186/s13068-016-0540-z (PMC4912819; doi:10.1186/s13068-016-0540-z)
Supplement: Supplementary file 1 — 10.1186/s12014-016-9116-y Variations of oriented fiber, microfibril angle, and axial coherence length across the stem for each mutant and wild type have been exhibited in figures S1–S8 of supplement material. [file 13068_2016_540_MOESM1_ESM.pdf]

Supplementary Material

1.1 Wild type sample (WT)

The fiber content of WT approached as high as ~25% in the xylem region. The microfibril angle slowly increased towards the center of the stem, a trend opposite that of fiber content. The (0 0 4) reflections are strong within the oriented part of the patterns and reflects a relatively constant coherence length of about ~210 Å at xylem. After storage in water for 30 days, fiber content is significantly reduced. Poorly oriented patterns frequently preclude measurement of microfibril angle and may have sufficiently weak (0 0 4) reflections to make measurement of coherence length impractical. Extended storage in water results in lowered fiber content and much wider variation of microfibril angle.

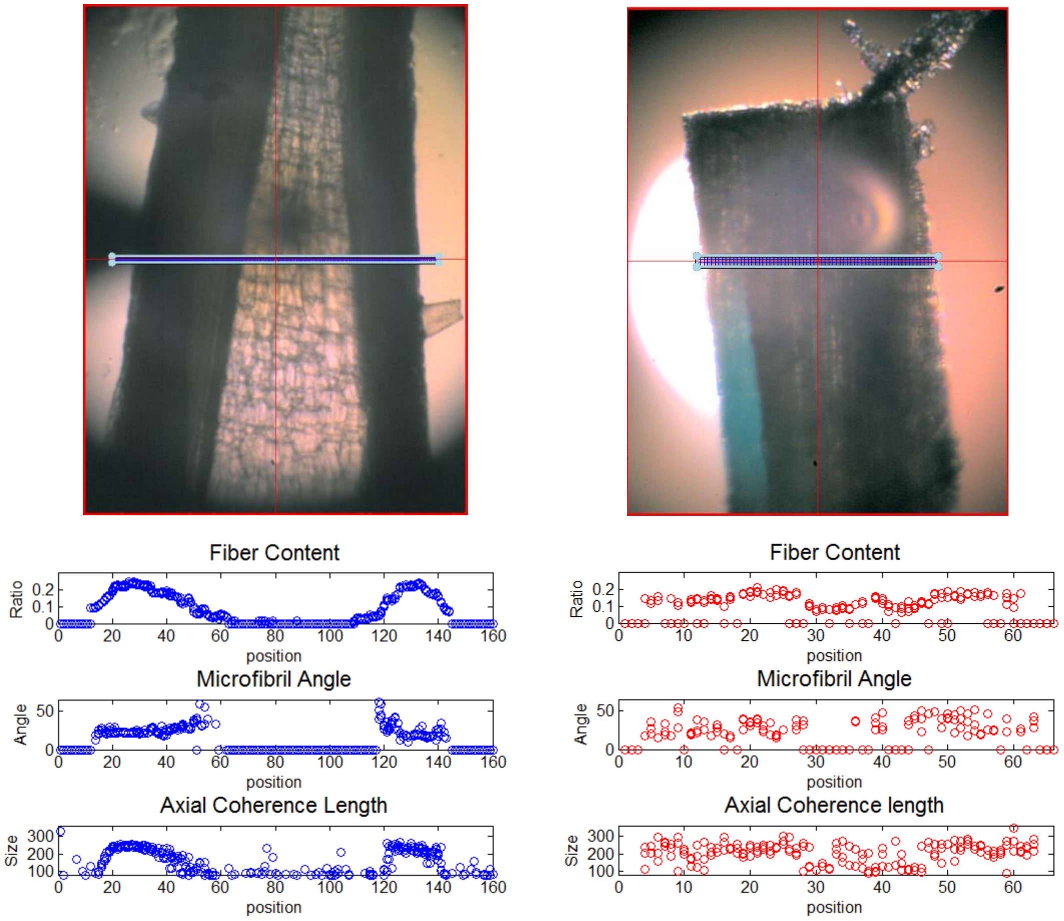

Figure S1. WT sample dried 3 (left) and 30 (right) days after harvest. Optical micrographs indicate the location of the microdiffraction scans. Fiber content, microfibril angle and axial coherence length are plotted as a function of position across the stem.

1.2 Aldehyde (G'S') sample (*cad-c cad-d*)

The fiber content of aldehyde at xylem could approach as high as ~25%. microfibril angle is observed across the stem with the opposite trend to the fiber content. The axial coherence length is, in general, smaller that WT, about ~196 Å at xylem. Storage in water results in a significant reduction of fiber content, to less than 10%. Even the best oriented patterns are usually too weak to make possible measurement of microfibril angle and coherence length. The overall impression is that cellulose fibrils are highly disordered and disoriented in all tissues within the stem.

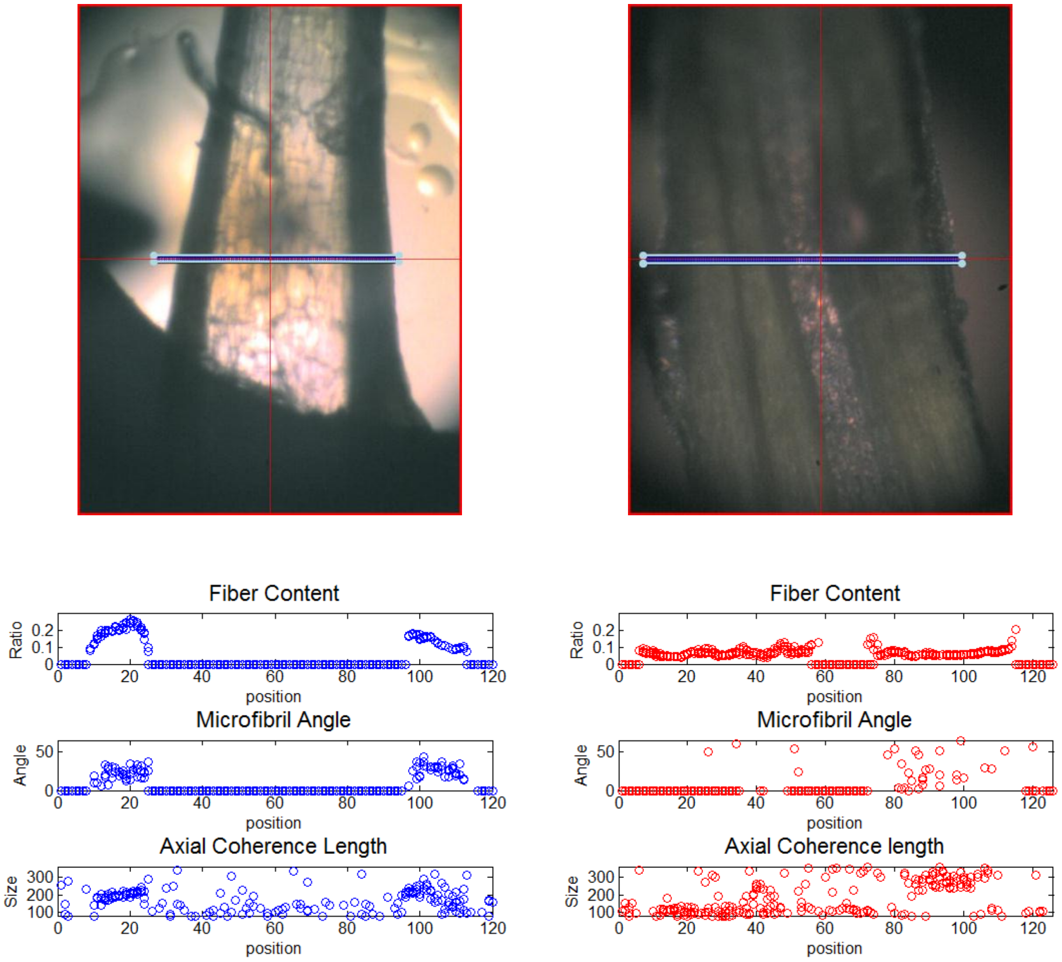

Figure S2. Aldehyde sample dried 3 (left) and 30 (right) days after harvest. Optical micrographs indicate the location of the microdiffraction scans. Fiber content, microfibril angle and axial coherence length are plotted as a function of position across the stem.

1.3 Aldehyde in G (G') sample (*cad-c cad-d fah1*)

The aldehyde in G (G') exhibits properties similar to those of the aldehyde sample. The highest fiber content of G' was ~25%. Microfibril angle is observed to vary modestly across the stem, generally exhibiting a trend opposite that of fiber content. The coherence length is, like the aldehyde samples, smaller than WT at about ~196Å in the xylem. After 30 days storage in water a significant reduction of fiber content is observed, down to less than 10%. The oriented patterns are again too weak to make possible measurement of microfibril angle or coherence length. The cellulose fibrils are highly disordered for the 30-day old sample. The thin sections of stem appeared collapsed in the optical micrographs (Figure S3) reflecting the dramatic impact of this chaotic arrangement of components on overall morphology.

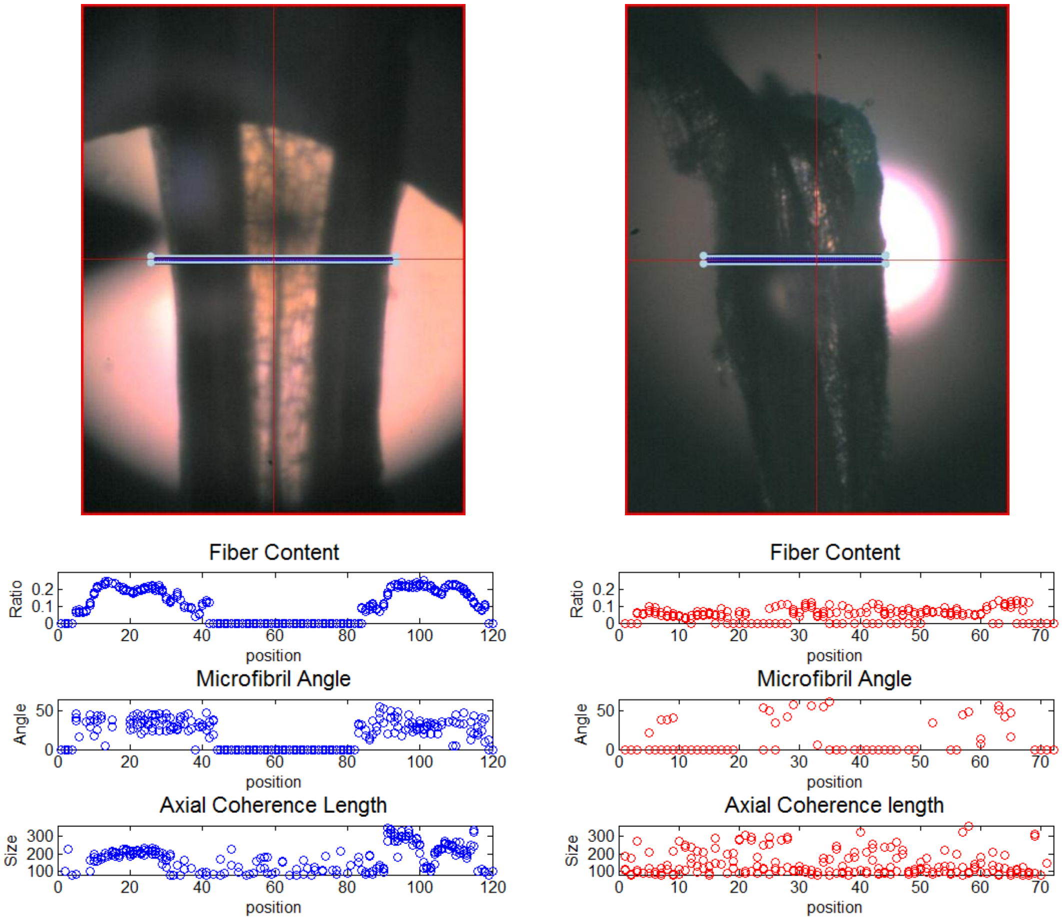

Figure S3. Aldehyde in G(G') sample dried 3 (left) and 30 (right) days after harvest. Optical micrographs indicate the location of the x-ray microdiffraction scans. Fiber content, microfibril angle and axial coherence length are plotted as a function of position across the stem.

1.4 High G sample (*fah1-2*)

Samples with high G lignin exhibit oriented fibril content comparable to WT at the xylem. As for WT samples, microfibril angle can be observed across the stem and exhibits the opposite trend of oriented fiber content. The (0 0 4) reflections are strong, allowing measurement of coherence length within oriented patterns, which is equal to ~220Å within the xylem. After 30 days storage, the fiber content decreased modestly, suggesting that G lignin may be efficient in protecting cellulose from degradative processes that led to severe disorder and disorientation in other samples.

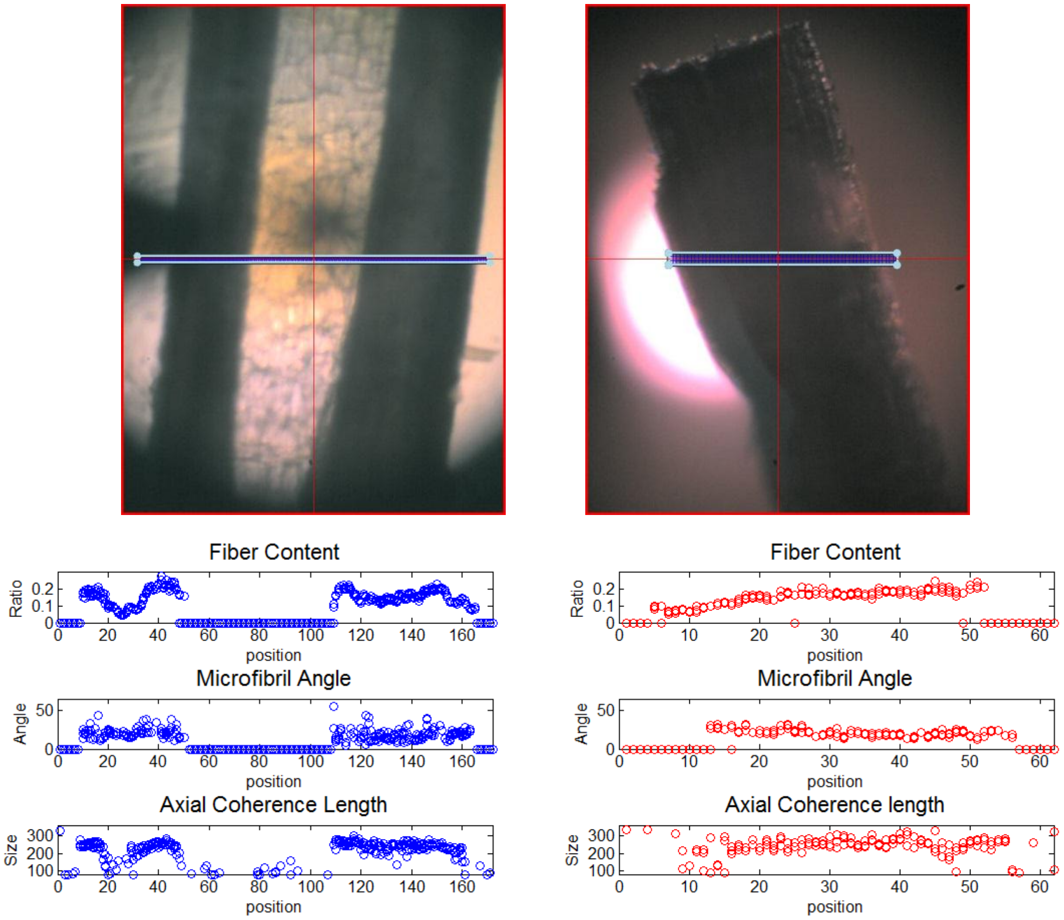

Figure S4. High G sample dried 3 (left) and 30 (right) days after harvest. Optical micrographs indicate the location of the x-ray microdiffraction scans. Fiber content, microfibril angle and axial coherence length are plotted as a function of position across the stem.

1.5 High S sample (*fah1-2/C4H:F5H*)

The highest oriented fiber content observed in high S samples was only ~22%. Microfibril angle is observed clearly across the stem, again exhibiting the opposite trend of oriented fiber content. The (0 0 4) reflections are strong and well defined and reflect a coherence length of ~210Å at xylem, about the same as WT. After 30 days storage, the fiber content decreases modestly, indicating that S-lignin, like G-lignin, may act to provide some resilience of cellulose fibrils to degradative processes.

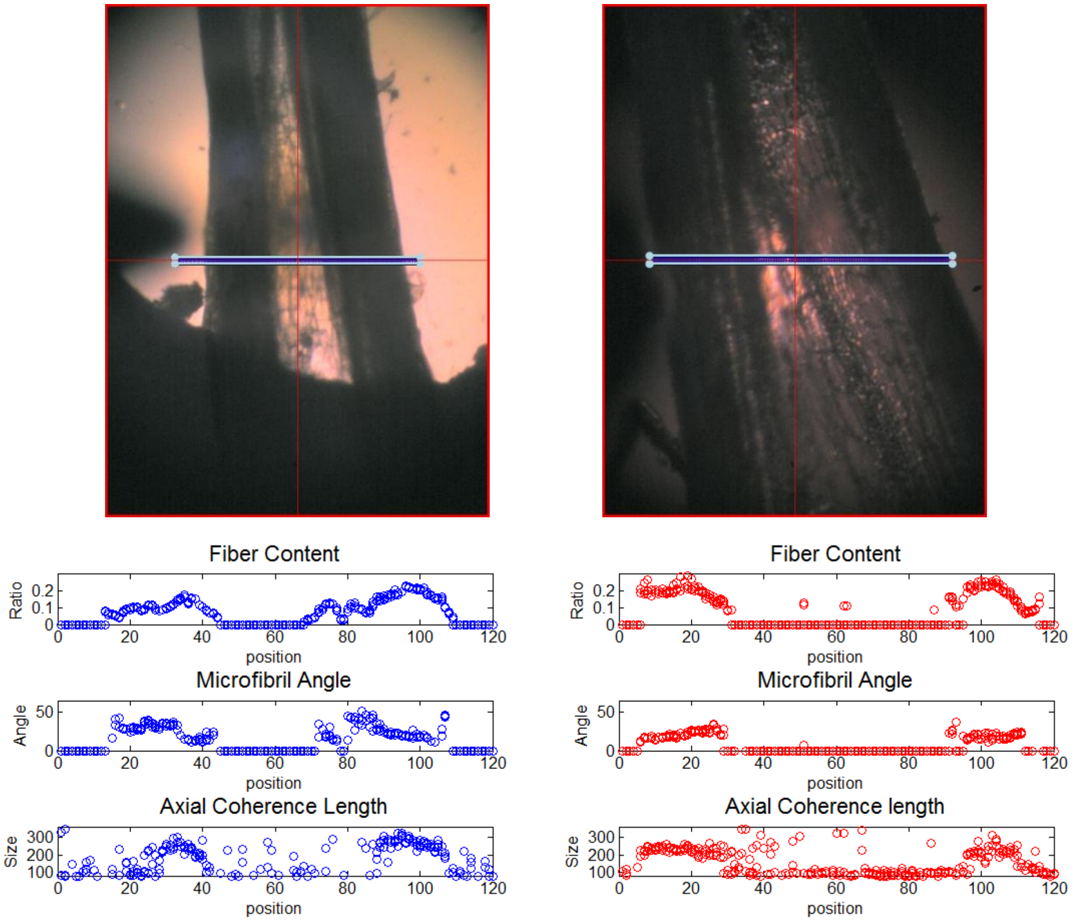

Figure S5. High S sample dried 3 (left) and 30 (right) days after harvest. Optical micrographs indicate the location of the x-ray microdiffraction scans. Fiber content, microfibril angle and axial coherence length are plotted as a function of position across the stem.

1.6 High H lignin sample (*ref4 rfr1 ref8*)

The fiber content of High H at xylem approaches ~25% similar to WT, . There is a distribution of microfibril angle across the stem with the opposite trend exhibited by oriented fiber content. The (0 0 4) reflection gives rise to a mean coherence length of about ~200Å, somewhat smaller than WT. After 30 days, structural degradation leads to significant reduction of oriented fiber content. The weaker oriented patterns in general preclude measurement of microfibril angle or coherent length. Qualitatively, the mutation gives rise to degradation of cellulose organization similar to that observed in the G' and S' mutants.

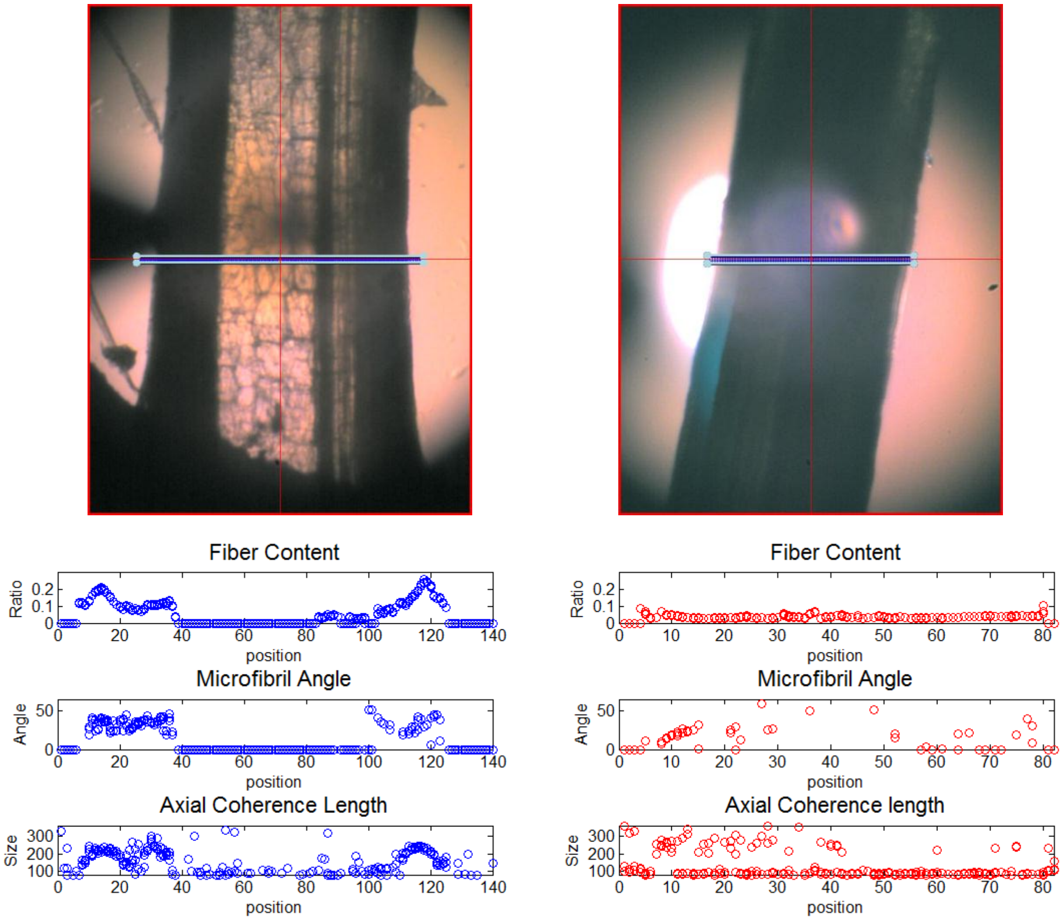

Figure S6. High H sample dried 3 (left) and 30 (right) days after harvest. Optical micrographs indicate the location of the x-ray microdiffraction scans. Fiber content, microfibril angle and axial coherence length are plotted as a function of position across the stem.

1.7 ferulate incorporated lignin sample (*ccr*)

The fiber content of *ccr* at xylem never exceeded ~19%, significantly lower than WT. Microfibril angle could be observed in almost all positions across the stem. The axial coherence length was ~210 Å at xylem, about the same as WT. After 30 days storage, oriented fiber content is reduced. The resulting weaker oriented patterns make measurement of microfibril angle difficult in many cases and decrease the mean coherence length, indications of significant decrease in order and orientation of the cellulose in these samples.

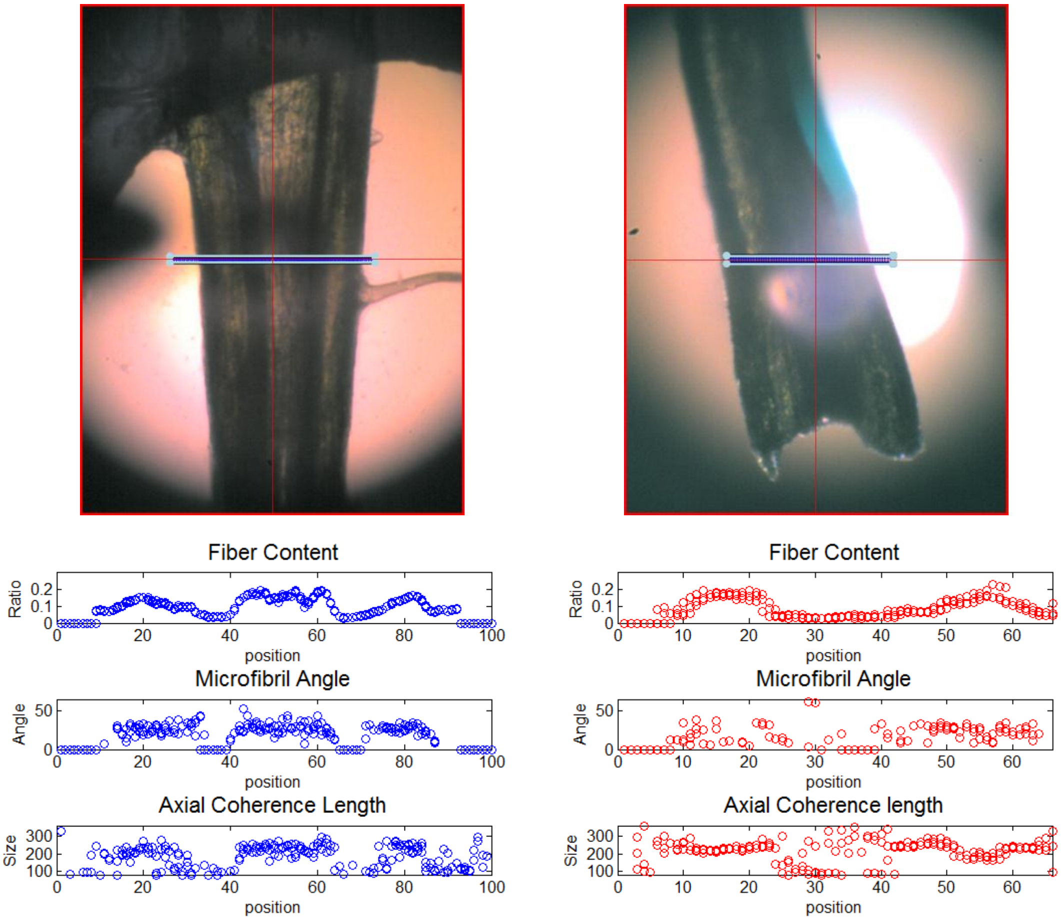

Figure S7. Ferulic acid-containing sample dried 3 (left) and 30 (right) days after harvest. Optical micrographs indicate the location of the x-ray microdiffraction scans. Fiber content, microfibril angle and axial coherence length are plotted as a function of position across the stem.

1.8 low lignin sample (*ref3-2*)

ref3-2 has much smaller fiber content compared to other mutants. Almost nowhere is orientation adequate to make possible measurement of microfibril angle. The coherence length is also significantly less than other samples, about ~160Å at xylem. After 30 days storage, there is a further reduction of fiber content, but in some samples the orientation was good enough to make possible measurement of microfibril angle. But most patterns were sufficeintly weak as to preclude measurement of microfibril angle or coherence length.

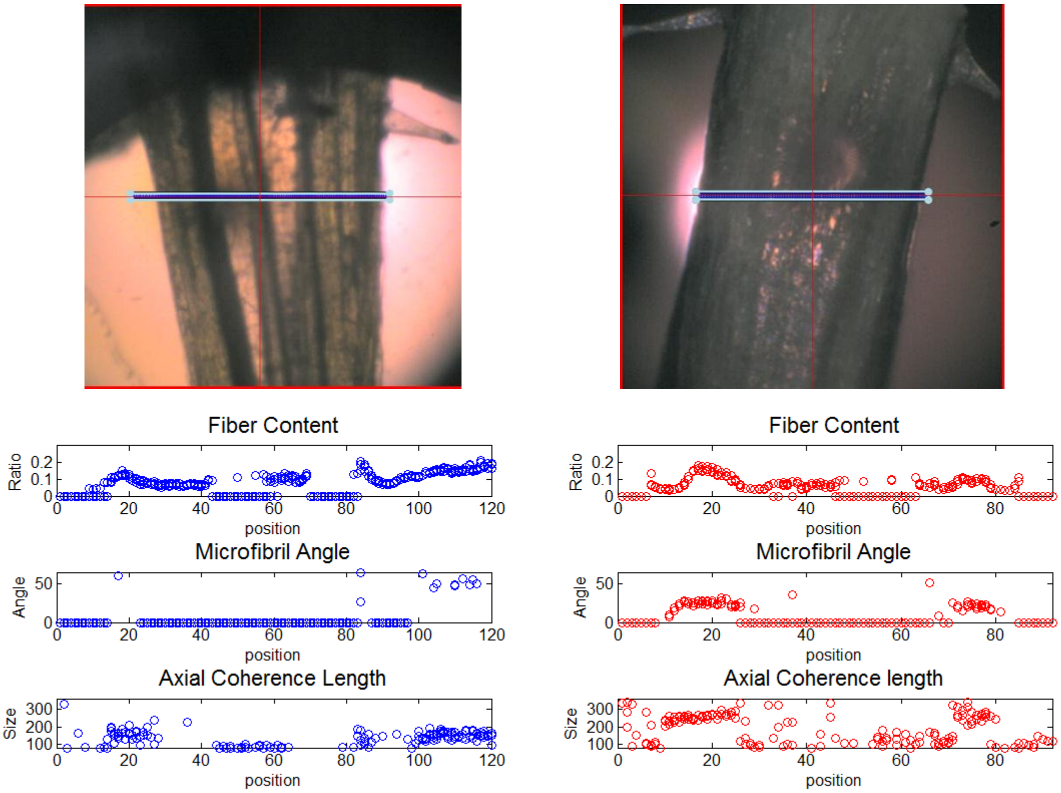

Figure S8. Low lignin sample dried 3 (left) and 30 (right) days after harvest. Optical micrographs indicate the location of the x-ray microdiffraction scans. Fiber content, microfibril angle and axial coherence length are plotted as a function of position across the stem.
